# High carbon dioxide emissions from Australian estuaries driven by geomorphology and climate

**DOI:** 10.1038/s41467-024-48178-4

**Published:** 2024-05-10

**Authors:** Jacob Z.-Q. Yeo, Judith A. Rosentreter, Joanne M. Oakes, Kai G. Schulz, Bradley D. Eyre

**Affiliations:** Centre for Coastal Biogeochemistry, Faculty of Science and Engineering, Southern Cross University, PO Box 157, East Lismore, NSW 2480 Australia

**Keywords:** Carbon cycle, Carbon cycle

## Abstract

Estuaries play an important role in connecting the global carbon cycle across the land-to-ocean continuum, but little is known about Australia’s contribution to global CO_2_ emissions. Here we present an Australia-wide assessment, based on CO_2_ concentrations for 47 estuaries upscaled to 971 assessed Australian estuaries. We estimate total mean (±SE) estuary CO_2_ emissions of 8.67 ± 0.54 Tg CO_2_-C yr^−1^, with tidal systems, lagoons, and small deltas contributing 94.4%, 3.1%, and 2.5%, respectively. Although higher disturbance increased water-air CO_2_ fluxes, its effect on total Australian estuarine CO_2_ emissions was small due to the large surface areas of low and moderately disturbed tidal systems. Mean water-air CO_2_ fluxes from Australian small deltas and tidal systems were higher than from global estuaries because of the dominance of macrotidal subtropical and tropical systems in Australia, which have higher emissions due to lateral inputs. We suggest that global estuarine CO_2_ emissions should be upscaled based on geomorphology, but should also consider land-use disturbance, and climate.

## Abstract (web summary)

Australian estuaries shown to emit more CO_2_ per unit area than global estuaries due to the dominance of macrotidal subtropical and tropical tidal systems, while disturbance effects were minimal due to low overall disturbance.

## Introduction

Estuaries play an important role connecting the carbon cycle across the land-to-ocean aquatic continuum, processing large amounts of allochthonous and autochthonous carbon^1^. This is despite estuaries constituting only a small fraction of the world’s surface (0.2%)^2^ compared to continental shelf seas (5%)^3^ and the open ocean (64%)^4^. Carbon from upstream rivers and associated coastal wetlands entering estuaries is either buried (and potentially stored long-term), emitted to the atmosphere in the form of greenhouse gases, or exported to the ocean^5,6^. Estuarine CO_2_ emissions are estimated to equate to the size of the CO_2_ sink in shelf seas (0.268 ± 0.225 Pg C yr^−1^), or 19% of the CO_2_ sequestration in the open ocean^7^, but early estimates were mostly based on studies in the northern hemisphere (e.g. refs. ^5,6,8,9^). CO_2_ emissions from estuaries can differ between estuary geomorphic types^10,11^, but the mechanisms by which geomorphology affects estuarine CO_2_ emissions in Australia have not been determined. There is also limited knowledge of how disturbance impacts CO_2_ emissions and how different geomorphic estuary types modify any disturbance effect.

Low to high disturbance and land-use changes in the upper catchment have the potential to alter the quantity and quality of carbon delivered to estuaries^5,12^, and hence the associated estuary CO_2_ emissions^12,13^. Dissolved inorganic carbon (DIC)^14^, dissolved organic carbon (DOC)^15^, and particulate organic carbon (POC)^16^ inputs typically increase in impacted estuaries and tend to be associated with increased CO_2_ emissions^7,17,18^. However, the effect of land-use on CO_2_ emissions from estuaries can vary^12,19^. For instance, moderately and highly disturbed estuaries in Australia have been reported to emit more CO_2_ per unit area (37 ± 10 mmol CO_2_-C m^−2^ d^−1^) than less disturbed estuaries (6.3 ± 4 mmol CO_2_-C m^−2^ d^−1^)^12^, whereas very small coastal estuaries with high land-use changes (>90% of catchment modified) had lower CO_2_ emissions than estuaries with low land-use changes (~21% of catchment modified)^15^. Moreover, changes in land-use along an estuarine gradient can influence nutrient cycling (e.g., decomposition) and change the quantity and quality (labile or refractory) of organic matter inputs^20–22^, resulting in increases or decreases in CO_2_ emissions between riverine-upstream, mid-estuary, and near-marine regions^23,24^.

Estuaries of different geomorphology are the result of varying influences of river discharge, tidal amplitude, and wave energy, which determine estuarine hydrological characteristics such as water depth, current velocity, and water residence times^2,25,26^. In turn, water depth and current velocity influence the gas transfer velocity (*k*) which controls the rate of CO_2_ emission from the water into the atmosphere^27–29^. Water residence times control CO_2_ emissions in estuaries by determining the direction and intensity of estuarine water-air CO_2_ fluxes^2,7^, because long water residence times allow for more carbon decomposition, resulting in increased DIC that can be emitted as CO_2_^7,28,30^. Shorter water residence times accelerate DOC export to the ocean, resulting in lower CO_2_ emissions^17,18^. Stratification of the water column can also influence CO_2_ emissions^31,32^. Photosynthetic CO_2_ uptake occurs in the surface layer, whereas CO_2_ respired in the bottom waters is isolated from atmospheric exchange at the surface^32–34^, resulting in an overall increase in CO_2_ partial pressure (*p*CO_2_) but unaffected water-air CO_2_ flux rates^32,34^. Stratification occurs in estuaries with weak tidal forcing, which leads to the separation of water layers of different densities (i.e. salinities)^31,35^ or temperatures (thermohaline stratification)^32–34^. In estuaries with stronger tidal influence, tidal pumping can also increase CO_2_ emissions through the lateral import of DIC and DOC from coastal wetlands to the estuary^36,37^. Tidal pumping can be a significant driver of CO_2_ emissions, with groundwater-derived export accounting for 93% to 99% of DIC and 89% to 92% of DOC exported from mangroves into tidal creeks^36^. In very large river systems^2^ such as the Amazon River^38^ in Brazil and the Yangtze^39^ River in China, riverine transport from the estuary to the ocean can result in extensive estuarine plumes that can act as either a source or a sink of CO_2_. However, such systems do not exist in Australia. A recent global analysis showed that fjords predominantly act as CO_2_ sinks, while tidal systems and deltas emit more CO_2_ than lagoons^11^.

Australia makes a significant contribution to the total number and surface area of estuaries globally. Australia’s coastline of 36,700 km includes 971 estuaries^40^, accounting for 1.82% of the total number of estuaries globally^41^ and 2.35% of global estuarine surface area^2^. More importantly, the majority of Australia’s estuaries (70.6%) are classified as low or moderately disturbed^40^, contrasting with predominantly disturbed estuaries in Europe and the United States where the majority of estuarine CO_2_ emissions have been measured^9,10^. This reflects Australia’s population density of only 3.3 persons km^−2^^42^, the 3rd lowest in the world. Despite Australia’s contribution to global estuary number and surface area, CO_2_ emissions have been measured in only a few Australian estuaries (e.g. refs. ^12,23,43^,), and there are no estimates of total CO_2_ emissions from Australian estuaries. In this study, we (1) calculated CO_2_ emissions from *p*CO_2_ measurements from 36 estuaries in different climate zones in Australia and combined these CO_2_ emission estimates with previously published CO_2_ emissions from 11 other Australian estuaries^12,30^ (total of 47 estuaries), and (2) assessed the interaction effects of anthropogenic disturbance and geomorphology on CO_2_ emissions for these 47 estuaries. Based on disturbance and geomorphology classifications of Australian estuaries, we then scaled up CO_2_ emissions from the 47 estuaries to all 971 assessed estuaries to better constrain Australia’s contribution to global estuarine CO_2_ emissions. We hypothesised that estuarine geomorphic type and disturbance level (including land-use change) would significantly impact estuarine water column *p*CO_2_ and CO_2_ emissions, and that there would be an interaction between geomorphic type, disturbance level, and CO_2_ emissions. We further hypothesised that relative CO_2_ emissions from Australian estuaries would be lower than global estuary CO_2_ emissions because of generally lower disturbance found in estuaries in Australia.

## Results

### Physical differences between estuary types

**Fig. 1:**
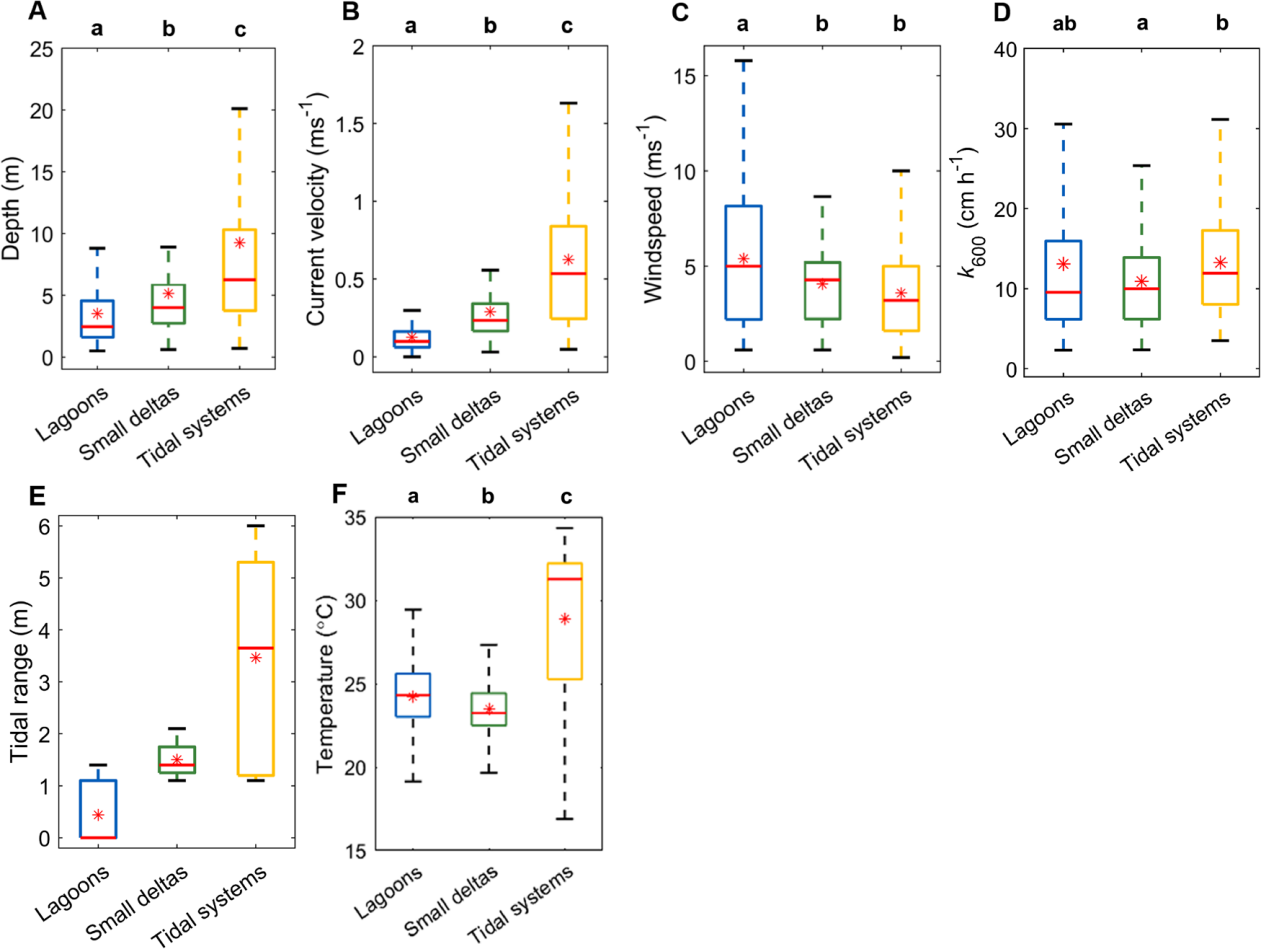
Physical parameters in the three estuary types. Median (red line), mean (red asterisk), 1st and 3rd interquartile ranges (box caps), minimum, and maximum values (whiskers) of (**A**) water depth, (**B**) current velocity, (**C**) wind speed, (**D**) mean gas transfer velocity normalised to Schmidt no. 600 (*k*_600_) calculated from the five parameterisations (Table 2), (**E**) tidal range, and (**F**) temperature in the lagoons (blue, *n*: **A** = 92, **B** = 101, **C** = 88, **D** and **F** = 3789, and **E** = 21), small deltas (green, *n*: **A** = 75, **B** = 67, **C** = 79, **D** = 3362, **E** = 12, and **F** = 3622), and tidal systems (yellow, *n*: **A** = 121, **B** = 112, **C** = 126, **D** = 5207, **E** = 14, and **F** = 5720). Outliers were omitted from the graphs. Letters above figures denote statistical differences among estuary types, with letters that are the same indicating no significant difference (PERMANOVA, two-tailed, and at 95% confidence interval). Source data are provided as a Source Data file.

Mean (min-max) tidal range was highest in tidal systems (*n* = 14) (3.7 m (1.1–6.0 m)), moderate in small deltas (*n* = 12) (1.5 m (1.1–2.1 m)), and generally lower in lagoons (*n* = 21) (0.6 m (0–1.4 m)) (Fig. 1E). Water depth (*n* = 92, 75, and 121, respectively) (Fig. 1A) and current velocity (*n* = 101, 67, and 112, respectively) (Fig. 1B) significantly increased (*p* = 0.001) from lagoons to small deltas to tidal systems. Wind speed was significantly higher in lagoons (*n* = 88) than in small deltas (*n* = 79) (*p* = 0.004) and tidal systems (*n* = 126) (*p* = 0.001), but was similar between small deltas and tidal systems (*p* = 0.137) (Fig. 1C). The mean gas transfer velocity normalised to the Schmidt number of 600 (*k*_600_), was highest in tidal systems and significantly lower in small deltas (*p* = 0.001). Although lagoons had the lowest *k*_600_ (*n* = 751), it was not significantly different from small deltas (*n* = 667) and tidal systems (*n* = 1036) (negative t-values) (Fig. 1D and Supplementary Fig. 1). Tidal range significantly increased from the lowest in lagoons (*n* = 21) to the highest in tidal systems (*n* = 14, small deltas: *n* = 12) (*p* = 0.001) (Fig. 1E). Temperature differed significantly between estuary types (lagoons: *n* = 751) (*p* = 0.001) with the lowest mean temperature in small deltas (*n* = 719) and the highest mean temperature in tidal systems (*n* = 1138) (Fig. 1F and Supplementary Table 3). Across all estuary types, except tidal systems, and within estuary types, temperature did not significantly correlate with *p*CO_2_ and water-air CO_2_ flux. In tidal systems, there was a significant increase in water-air CO_2_ flux with temperature (*r* = 0.254, *p* = 0.01).

### Estuary *p*CO_2_ and water-air CO_2_ fluxes

**Fig. 2:**
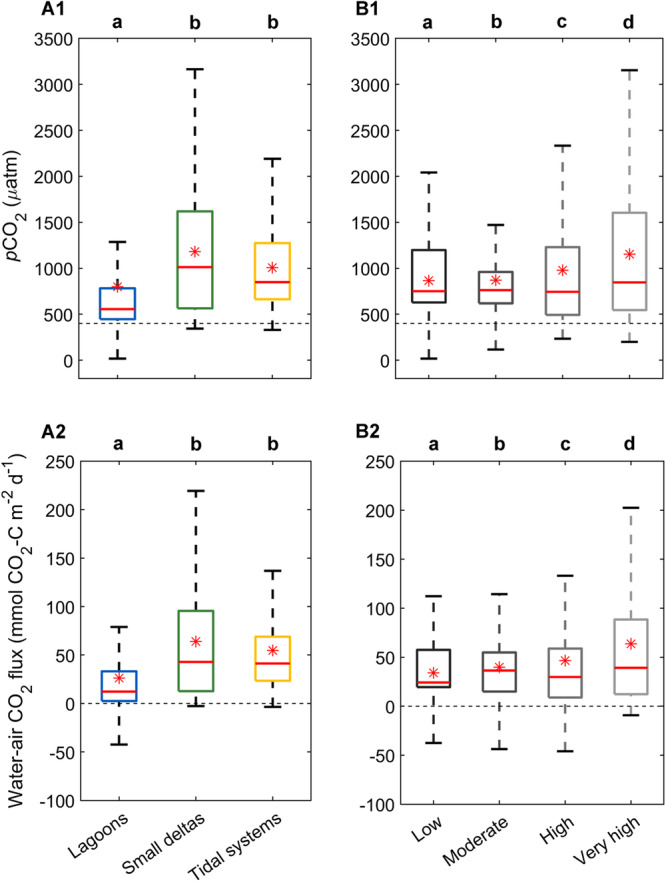
CO_2_ partial pressure (*p*CO_2_) and water-air CO_2_ flux in estuary types and disturbance groups. Median (red line), mean (red asterisk), 1st and 3rd interquartile ranges (box caps), minimum and maximum values (whiskers) of *p*CO_2_ and water-air CO_2_ flux at per-minute resolution in the (**A**) lagoons (blue, *n* = 3789), small deltas (green, *n* = 3622), and tidal systems (yellow, *n* = 5720); and (**B**) low (black, *n* = 1796), moderate (dark grey, *n* = 3189), high (grey, *n* = 3677), and very high (light grey, *n* = 4469) disturbance groups. Outliers were omitted from the figures. Dotted line along the x-axis represents atmospheric *p*CO_2_ and water-air flux CO_2_ equilibrium. Letters above figures denote statistical differences among estuary types, with letters that are the same indicating no significant difference (PERMANOVA, two-tailed, and at 95% confidence interval). Source data are provided in the Source Data file.

The majority of the estuaries studied were a source of CO_2_ to the atmosphere (Fig. 2A). Mean (±SE) *p*CO_2_ and water-air CO_2_ fluxes were 799 ± 13 µatm and 26.4 ± 0.9 mmol CO_2_-C m^−2^ d^−1^ in lagoons (*n* = 751), 1181 ± 12 µatm and 63.9 ± 1.1 mmol CO_2_-C m^−2^ d^−1^ in small deltas (*n* = 719), and 1007 ± 6 µatm and 54.8 ± 0.8 mmol CO_2_-C m^−2^ d^−1^ in tidal systems (*n* = 1138) (Table 1). Six small deltas and three tidal systems had small sections that were weak CO_2_ sinks (>−3.5 mmol CO_2_-C m^−2^ d^−1^). Four lagoons were overall CO_2_ sinks, whereas 14 lagoons had sections that were strong CO_2_ sinks (up to −64.7 mmol CO_2_-C m^−2^ d^−1^). Although *p*CO_2_ and water-air CO_2_ fluxes in the lagoons had the largest range, *p*CO_2_ and water-air CO_2_ fluxes were significantly lower than in small deltas and tidal systems (*p* = 0.001), with lower means and medians (Fig. 2A). *p*CO_2_ (*p* = 0.693) and water-air CO_2_ fluxes (*p* = 0.064) in small deltas were not significantly different to those in tidal systems (Fig. 2A).

**Table 1:** Descriptive statistics calculated for *p*CO_2_ and water-air CO_2_ fluxes using data at per-minute resolution of each estuary type, disturbance group, and in the disturbance groups within each estuary type

		*p*CO_2_ (μatm)						Water-air CO_2_ flux (mmol CO_2_-C m^−2^ d^−1^)					
		Per-minute (averaged per-estuary)						Per-minute (averaged per-estuary)					
Estuary type	Disturbance	Mean	Median	SE	IQR	Min	Max	Mean	Median	SE	IQR	Min	Max
Lagoons	All	799	555	13	336	17 (334)	9478 (1906)	26.4	12.4	0.9	30.5	−64.7 (−12.6)	546.7 (85.6)
Small deltas	All	1181	1012	12	1054	344 (413)	4342 (2703)	63.9	42.9	1.1	82.8	−2.6 (2.1)	401.1 (234.1)
Tidal systems	All	1007	849	6	611	329 (585)	2906 (1611)	54.8	41.3	0.8	45.4	−3.5 (11.8)	570.8 (131.8)
All	Low	864	751	10	569	17 (457)	2162 (855)	34.1	24.2	0.8	38.0	−46.2 (0)	172.5 (48.5)
All	Moderate	869	762	9	341	116 (454)	9478 (1927)	39.7	36.4	0.6	39.9	−64.7 (−5.5)	193.8 (75.4)
All	High	977	744	11	737	233 (405)	5371 (2238)	46.6	29.8	1.1	49.9	−46 (−3)	546.7 (183.9)
All	Very high	1152	846	12	1058	199 (417)	5791 (2454)	63.8	39.1	1.2	75.9	−9.2 (2)	570.8 (187.8)
Lagoons	Low	185	163	10	285	17 (92)	436 (203)	−18.7	−18.0	0.6	5.6	−46.2 (−29)	4 (−9.5)
	Moderate	657	559	28	194	116 (378)	9478 (1980)	10.1	9.2	0.8	9.9	−64.7 (−16.9)	193.8 (48.3)
	High	859	536	22	509	233 (337)	5371 (2357)	37.7	20.7	2.0	37.8	−46 (−11.3)	546.7 (177.7)
	Very high	916	604	23	377	199 (403)	5791 (2297)	31.9	14.2	1.2	35.8	−9.2 (-0.6)	233.8 (99.7)
Small deltas	High	1063	842	16	904	350 (410)	3992 (2535)	57.2	37.1	1.6	69.9	−2.1 (0.5)	401.1 (230.9)
	Very high	1296	1245	17	1113	344 (417)	4342 (2870)	70.3	51.2	1.6	96.3	−2.6 (3.7)	374.5 (237.3)
Tidal systems	Low	956	840	9	609	577 (731)	2162 (1344)	41.3	29.1	0.7	38.2	10.9 (21.7)	172.5 (92)
	Moderate	942	822	8	411	329 (562)	2846 (1854)	49.9	43.0	0.6	30.4	−3.5 (10.6)	164.1 (113.3)
	High	981	934	17	647	406 (558)	1683 (1052)	34.6	34.2	1.0	27.4	−0.1 (7.5)	114.8 (58.2)
	Very high	1217	1067	20	1017	397 (445)	2906 (1937)	92.8	72.2	3.0	85.1	−2.4 (3.7)	570.8 (264.8)

The mean of minimum and maximum values calculated for each estuary are presented in brackets.

*SE* standard error, *IQR* interquartile range (3rd quartile–1st quartile).

### Disturbance effects on estuary CO_2_

**Fig. 3:**
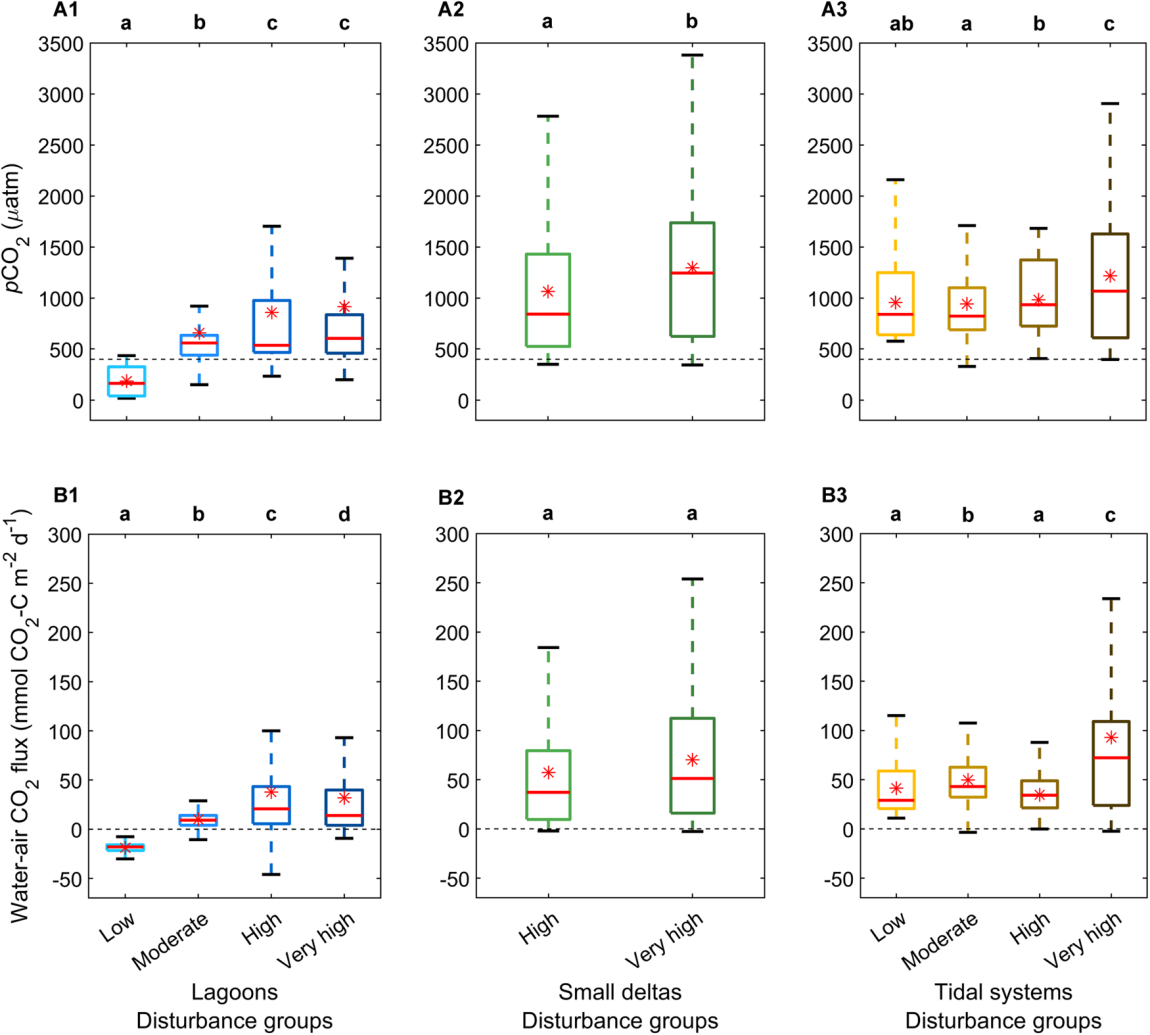
CO_2_ partial pressure (*p*CO_2_) and water-air CO_2_ flux in estuary type disturbance groups. Median (red line), mean (red asterisk), 1st and 3rd interquartile ranges (box caps), minimum and maximum values (whiskers) of (row A) *p*CO_2_, and (row B) water-air CO_2_ flux at per-minute resolution across different disturbance groups within (column 1) lagoons (from low (light blue) to very high disturbance (dark blue), *n* = 214, 815, 1312, and 1448), (column 2) small deltas (*n*; high (light green)=1777, and very high (dark green)=1845), and (column 3) tidal systems (from low (light yellow) to very high disturbance (dark brown), *n* = 1582, 2374, 588, and 1176). Outliers were omitted from the figures. Dotted line along *x*-axis represents atmospheric *p*CO_2_ and water-air CO_2_ flux equilibrium. Letters above figures denote statistical differences among estuary types, with letters that are the same indicating no significant difference (PERMANOVA, two-tailed, and at 95% confidence interval). Source data are provided in the Source Data file.

*p*CO_2_ and water-air CO_2_ fluxes significantly increased with greater disturbance in Australian estuaries (low to very high disturbance, n = 356, 633, 731, and 888, respectively) (Fig. 2B). For example, mean water-air CO_2_ fluxes across all estuary types increased from 34.1 ± 0.8 mmol CO_2_-C m^−2^ d^−1^ in the low disturbance systems to 63.8 ± 1.2 mmol CO_2_-C m^−2^ d^−1^ in the very high disturbance systems (Table 1). However, the effect of disturbance on *p*CO_2_ and water-air CO_2_ fluxes was estuary type specific (Fig. 3). In the lagoons, *p*CO_2_ in the low disturbance systems (*n* = 41) was below atmospheric equilibrium (Fig. 3A1), with CO_2_ influx from the atmosphere into the estuarine waters (range: 17 to 436 µatm, −46.2 to 4 mmol CO_2_-C m^−2^ d^−1^; Figure 3A2). *p*CO_2_ increased significantly in higher disturbance lagoons (*p* = 0.001), except between highly and very highly disturbed lagoons (*p* = 0.934) (moderate to very high disturbance, *n* = 161, 261, and 288, respectively). Similarly, water-air CO_2_ flux in lagoons increased significantly with higher disturbance (*p* = 0.001), but only from low to high disturbance, and was significantly lower in very high disturbance lagoons compared to high disturbance lagoons (*p* = 0.037).

In the small deltas, *p*CO_2_ was significantly higher in the very high disturbance systems (*n* = 366) compared to the high disturbance systems (*n* = 353) (*p* = 0.001), but water-air CO_2_ flux was similar between the high and very high disturbance systems (*p* = 0.101) (Fig. 3B). No measurements were taken in low and moderate disturbance small deltas. In the tidal systems, disturbance effects on *p*CO_2_ were insignificant in the low (*n* = 315) and moderate (*n* = 472) (*p* = 0.682), and low and high (*n* = 117) disturbance systems (*p* = 0.118) but significantly increased from the moderate to high disturbance systems (*p* ≥ 0.006) (Fig. 3C). *p*CO_2_ in the very high (*n* = 234) disturbance tidal systems was significantly greater than in low disturbance systems (*p* = 0.001). Water-air CO_2_ fluxes significantly increased with higher disturbance (*p* = 0.001) except between the low and high disturbance systems (*p* = 0.094). Water-air CO_2_ fluxes were greatest in very high disturbance systems (Fig. 3C2 and Table 1).

### Seasonal CO_2_ emissions from Australian estuaries

We estimated that Australian estuaries emit a mean (±SE) of 7.62 ± 0.48 Tg CO_2_-C yr^−1^ over the summer season, of which tidal systems contributed 93.4%, and lagoons and small deltas contributed 4.4% and 2.2%, respectively. To estimate winter water-air CO_2_ fluxes, seasonal ratios from published summer and winter water-air CO_2_ fluxes from 13 estuaries (Supplementary Table 1) were averaged to obtain the seasonal ratio (winter CO_2_ flux: summer CO_2_ flux) for each estuary type (Supplementary Table 2) which were then applied to summer water-air CO_2_ fluxes from the current study. In these 13 estuaries, lagoons in winter had a lower mean CO_2_ uptake, with seasonal ratios ranging from 0.58 to 0.69 (mean: 0.64; Supplementary Table 2). Small deltas and tidal systems in winter had higher mean water-air CO_2_ flux rates than in summer, with seasonal ratios ranging from 0.33 to 4.71 in small deltas (mean: 1.49) and 0.43 to 2.17 in tidal systems (mean: 1.3) (Supplementary Table 2). Winter water-air CO_2_ fluxes in Australian estuaries had a mean (±SE) flux rate of 49.7 ± 7.5 mmol CO_2_-C m^−2^ d^−1^, 25.8% higher than summer flux rates (Table 2).

**Table 2:** Mean, standard error (SE), and median for summer, winter, and annual water-air CO_2_ flux rates and annual CO_2_ emissions from Australian estuaries (Estuary (Est.) types: La: lagoons, SD: small deltas, TS: tidal systems; Disturbance (Dist.) groups: 1: low, 2: moderate, 3: high, 4: very high)

		Summer			Winter					Annual (Summer + Winter)									
		Water-air CO_2_ flux (mmol CO_2_-C m^−2^ d^−1^)			Water-air CO_2_ flux (mmol CO_2_-C m^−2^ d^−1^)					Water-air CO_2_ flux (mmol CO_2_-C m^−2^ d^−1^)					Australian CO_2_ emissions (Tg CO_2_-C yr^−1^)				
Est. type	Dist.	Median	Mean	SE	Median	Mean	SE	Mean (−)	Mean (+)	Median	Mean	SE	Mean (−)	Mean (+)	Median	Mean	SE	Mean (−)	Mean (+)
La																			
	1	−18.3	−18.8	0.9	−11.6	−12.0	0.6	−11.0	−13.0	−15.0	−15.4	0.8	−14.9	−15.9	−0.02	−0.02	0.00	−0.02	−0.02
	2	9.9	12.5	5.2	6.3	7.9	3.3	7.3	8.6	8.1	10.2	4.3	9.9	10.5	0.01	0.01	0.01	0.01	0.01
	3	19.5	30.4	10.8	12.4	19.3	6.9	17.7	20.9	16.0	24.8	8.8	24.0	25.6	0.05	0.08	0.03	0.07	0.08
	4	21.1	26.8	7.4	13.4	17.0	4.7	15.6	18.5	17.2	21.9	6.1	21.2	22.6	0.16	0.20	0.06	0.19	0.21
	All	12.8	16.3	5.0	8.1	10.4	3.2	9.5	11.3	10.5	13.4	4.1	12.9	13.8	0.20	0.27	0.05	0.26	0.28
SD																			
	1^a^	–	–	–	–	–	–	–	–	–	–	–	–	–	0.03	0.03	–	0.02	0.08
	2^a^	–	–	–	–	–	–	–	–	–	–	–	–	–	0.03	0.03	–	0.02	0.07
	3	51.4	58.3	14.7	76.8	87.1	22.0	19.2	275.0	64.1	72.7	18.3	38.8	166.7	0.03	0.04	0.01	0.02	0.08
	4	61.9	70.2	10.7	92.5	104.8	15.9	23.1	330.7	77.2	87.5	13.3	46.6	200.4	0.10	0.11	0.02	0.06	0.26
	All	58.8	64.2	8.8	87.9	95.9	13.2	21.2	302.9	73.3	80.1	11.0	42.7	183.6	0.19	0.21	0.03	0.11	0.49
TS																			
	1	44.1	46.1	12.4	57.4	60.0	16.1	19.7	100.3	50.7	53.1	14.2	32.9	73.2	2.58	2.70	0.72	1.67	3.72
	2	50.0	51.8	4.0	65.1	67.4	5.2	22.2	112.7	57.6	59.6	4.6	37.0	82.3	4.33	4.48	0.35	2.78	6.19
	3	22.2	22.2	21.8	28.9	28.9	28.4	9.5	48.3	25.6	25.6	25.1	15.9	35.3	0.63	0.63	0.62	0.39	0.87
	4	72.1	84.4	42.6	93.8	109.8	55.4	36.1	183.5	82.9	97.1	49.0	60.2	134.0	0.21	0.25	0.13	0.15	0.34
	0^b^	–	–	–	–	–	–	–	–	–	–	–	–	–	0.11	0.12	0.02	0.08	0.17
	All	49.1	52.9	10.2	63.9	68.9	13.3	22.6	115.1	56.5	60.9	11.8	37.8	84.0	7.86	8.18	0.91	5.08	11.29
All est.		36.6	39.5	5.3	35.3	49.7	7.5	16.4	116.7	31.2	44.6	6.4	27.9	78.1	8.25	8.67	0.54	5.45	12.06
All	1	23.3	18.3	14.7	30.3	29.2	16.9	6.6	51.8	26.8	23.7	15.8	12.4	35.0	1.41	1.25	0.83	0.65	1.84
All	2	29.6	28.9	6.7	18.8	32.7	9.3	13.5	52.0	24.2	30.8	7.9	21.2	40.4	1.86	2.37	0.61	1.63	3.11
All	3	36.6	42.0	9.1	44.3	52.1	14.1	17.1	142.4	40.2	47.1	11.4	29.6	92.2	1.14	1.33	0.32	0.84	2.61
All	4	51.0	55.7	10.8	65.0	70.7	16.3	22.7	176.4	54.3	63.2	13.5	39.2	116.0	0.70	0.82	0.17	0.51	1.51

Descriptive statistics for summer water-air CO_2_ fluxes were calculated with per-minute resolution data measured in this study while winter water-air fluxes were calculated based on seasonal ratios. Up-adjusted means (+) (using maximum seasonal ratios) and down-adjusted means (−) (using minimum seasonal ratios) calculated from the sensitivity analysis are shown for winter and annual water-air CO_2_ fluxes and annual CO_2_ emissions.

^a^Mean and median low and moderate disturbance small delta annual CO_2_ emissions were calculated using mean and median small delta water-air CO_2_ flux rates.

^b^Mean and median annual CO_2_ emissions from tidal systems with disturbance classified no assessment were calculated using mean and median tidal system water-air CO_2_ flux rates.

### Annual CO_2_ emissions from Australian estuaries

The mean annual CO_2_ emission from Australian estuaries was 8.67 ± 0.54 Tg CO_2_-C yr^−1^ (Table 2), with tidal systems accounting for 94.4% of annual CO_2_ emissions, followed by small deltas at 2.5%, and lagoons at 3.1%. These proportions compared with surface areas for tidal systems, lagoons, and small deltas, representing 89.9%, 8.6%, and 1.5% of total Australian estuary surface area, respectively (Table 3). Due to the larger surface area coverage of lagoons with increased disturbance (Table 3), CO_2_ emissions from lagoons were dominated by the higher disturbance systems. High disturbance lagoons had the greatest CO_2_ flux rates, but very high disturbance lagoons covered a greater proportion of lagoon surface area (62%) and therefore, as a category, emitted the most CO_2_. In contrast, lower disturbance small deltas and tidal systems covered the largest proportion of their respective estuary-type surface area and emitted the most CO_2_ annually (Tables 2 and 3). Low and moderately disturbed tidal systems had the greatest total emissions, driven mainly by the large surface area coverage (33% and 48%, respectively) in remote northern Australia. Very high disturbance small deltas had the highest water-air fluxes and the largest proportion of total small delta surface area (50%) and therefore, emitted the most CO_2_ annually. Low disturbance lagoons were the only CO_2_ sinks of all the estuary types and disturbance groups, whereas in tidal systems the very high disturbance systems emitted the least CO_2_ annually. The low, moderate, and high disturbance small deltas emitted similarly low levels of CO_2_ annually (Table 2). Moderately disturbed Australian estuaries had the largest CO_2_ emissions, followed by the high, low, and very high disturbance systems (Table 2).

**Table 3:** Number of estuaries, estuarine surface area, and percent of total estuary surface area classified by estuary type according to Dürr et al.^2^ and classified by disturbance in NLWRA^40^ for sampled estuaries in this study and in all Australia

		Study surface area coverage			National surface area coverage		
Estuary type	Disturbance	Estuaries (*n*)	(km^2^)	% National representation	Estuaries (*n*)	(km^2^)	% Estuary type
Lagoons	Low	3	38	13.3	78	286	8.4
	Moderate	7	95	31.0	75	308	9.1
	High	5	226	32.1	82	704	20.8
	Very high	6	286	13.7	36	2083	61.6
	Not assessed	0	0	–	2	0	–
	Total	21	645	19.1	273	3382	8.6
Small deltas	Low	0	0	0.0	38	99	16.7
	Moderate	0	0	0.0	39	85	14.4
	High	6	18	16.3	47	112	18.9
	Very high	6	100	34.0	25	295	49.9
	Not assessed	–	–	–	0	0	–
	Total	12	119	20.1	149	591	1.5
Tidal systems	Low	4	1012	8.7	359	11598	32.7
	Moderate	5	1350	11.6	97	17152	48.4
	High	2	1553	13.4	61	5630	15.9
	Very high	3	179	1.5	27	582	1.6
	Not assessed	0	0	–	5	455	1.3
	Total	14	4094	11.6	549	35417	89.9
Total		47	4858	12.3^a^	971	39390	100
Disturbance	Estuary type	Estuaries (*n*)	(km^2^)	% National representation	Estuaries (*n*)	(km^2^)	% Disturbance group
Low	Lagoons			0.3			2.4
	Small deltas			0			0.8
	Tidal systems			8.4			96.8
	Total	7	1050	8.8	475	11983	30.4
Moderate	Lagoons			0.5			1.8
	Small deltas			0			0.5
	Tidal systems			7.7			97.8
	Total	12	1446	8.2	211	17545	44.5
High	Lagoons			3.5			10.9
	Small deltas			0.3			1.7
	Tidal systems			24.1			87.3
	Total	13	1797	27.9	190	6446	16.4
Very high	Lagoons			9.7			70.4
	Small deltas			3.4			10.0
	Tidal systems			6.0			19.7
	Total	15	565	19.1	88	2961	7.5
Not assessed	Lagoons			–			0.1
	Small deltas			–			0
	Tidal systems			0			99.9
	Total	–	–	–	7	455	1.2
Total		47	4858	12.3	971	39390	100

Two lagoons and five tidal systems were not assessed for disturbance^40^.

^a^Total represented coverage of the national estuarine surface area.

## Discussion

There was a strong geomorphic effect on measured *p*CO_2_ and water-air CO_2_ fluxes in Australian estuaries (Fig. 2A1 and A2), with the lagoons particularly different from the small deltas and tidal systems. Overall, lagoons had the lowest *p*CO_2_ and water-air CO_2_ fluxes of the three geomorphic types. This was likely driven by higher benthic productivity, which can result in a net autotrophic system with CO_2_ uptake across the water-air interface^43,44^ over a diurnal period^45,46^. Indeed, seagrass meadows cover an average of 18% of lagoon water areas in NSW, compared to only ~6% in small deltas and tidal systems^47^. Consistent with this, CO_2_ undersaturation and CO_2_ uptake have been reported in three Australian lagoons^43^, as well as non-Australian marine-dominated shallow coastal systems with a large cover of benthic vegetation (e.g. refs. ^44,48,49^). Freshwater input could also be a driver of *p*CO_2_ and water-air CO_2_ fluxes in estuaries; freshwater is typically supersaturated with CO_2_ and a source of allochthonous organic matter^5,50–52^ that may subsequently decompose and release CO_2_^7,51^. However, we found poor relationships between salinity and *p*CO_2_ and water-air CO_2_ fluxes in Australian lagoons (Supplementary Fig. 2), suggesting that freshwater organic matter was not an important source of CO_2_ in these systems. This may reflect a weak hydrological connection between lagoons and upstream rivers, which would limit input of riverine water. This is consistent with a previous study showing lower CO_2_ emissions in estuaries with lower riverine input compared to river-dominated estuaries^53^.

*p*CO_2_ and DIC concentration were higher in small deltas and tidal systems compared to lagoons (Figure 2A1, Supplementary Fig. 4B1, Supplementary Results). The inverse relationships between salinity and *p*CO_2_ or water-air CO_2_ fluxes in small deltas and tidal systems indicate that CO_2_ outgassing from the re-mineralisation of organic matter in upstream waters had a larger contribution in these systems compared to the lagoons^31^, as seen in other estuaries with higher riverine input^53^. This is because increasing *p*CO_2_ and water-air CO_2_ fluxes with decreasing salinity indicate that increasing CO_2_ is linked to freshwater input upstream. The tidal systems and small deltas also have a stronger connection to the river and associated input of CO_2_ supersaturated water^2^, which enhances CO_2_ emissions in the estuary. In our study, all of the small deltas and tidal systems were tropical and sub-tropical (23.5° to 35° latitude) (Supplementary Data 1), where much of the atmospheric carbon uptake and sequestration occurs within their mangrove-lined shorelines^54,55^. Lateral export from vegetated shorelines into adjacent estuaries can be a significant pathway for the transport of carbon in the form of DOC, POC, DIC, and CO_2_-rich pore water, or as a result of the degradation of exported organic matter^56–58^. Increased tidal range resulted in an increase in *p*CO_2_ and water-air CO_2_ fluxes (Supplementary Results), suggesting increased *p*CO_2_ and water-air CO_2_ fluxes were due to lateral export in our estuaries^56–58^, although we did not directly measure lateral inputs. Although estuaries in lower latitudes have higher water temperatures that could drive increased water-air CO_2_ fluxes, water temperature did not correlate with *p*CO_2_ and water-air fluxes in our estuaries (Supplementary Results). As such, higher *p*CO_2_ and DIC concentrations in small deltas and tidal systems compared to lagoons could likely be attributed to increased lateral inorganic (and organic) carbon export from intertidal coastal wetlands due to stronger lateral exchange by tides compared to lagoons (Fig. 1E).

In contrast to the lagoons, where DIC was positively correlated with *p*CO_2_ and water-air CO_2_ fluxes, in tidal systems and small deltas *p*CO_2_ and CO_2_ fluxes were not strongly associated with DIC concentrations; only CO_2_ fluxes in tidal systems showed a very weak trend with DIC concentration (Supplementary Results). Removing the effect of salinity in our analysis (as a co-variate), the differences in *p*CO_2_ and water-air CO_2_ flux correlations with DIC between estuary types suggests that other factors specific to small deltas and tidal systems further influence CO_2_ and DIC dynamics along the estuarine gradient in those systems. For example, shorter water residence times and increased intertidal wetlands are among factors driving processes impacting CO_2_ and DIC, which may include CO_2_ emissions of mangrove porewater DIC and mineralisation of exported POC and DOC^55,59,60^. The positive relationship between *p*CO_2_ and DOC (Supplementary Results and Supplementary Table 5) suggests that DOC mineralisation may drive increased *p*CO_2_ in small deltas. In tidal systems, stronger tidal influence compared to small deltas could further promote CO_2_ emissions through the tidal resuspension of sediments, releasing DIC and organic matter for remineralisation^61–63^, with excess DIC and organic matter exported to the coastal ocean^58,64^.

The sensitivity analysis showed that summer:winter water-air CO_2_ flux ratios ranged from 0.33 to 4.71 across the 13 estuaries (Supplementary Table 1). The largest range in summer:winter water-air flux CO_2_ ratios was for small deltas, where ratios were up to 3x larger than in lagoons and tidal systems (Supplementary Table 2). However, when estimating annual emissions, the large range in ratios in small deltas was attenuated by the large surface area of tidal systems compared to the small surface area coverage by small deltas (Table 3). Including mean winter water-air flux rates in annual Australian estuarine CO_2_ emission calculations only showed 13% greater water-air flux rate and 14% greater annual emissions than from summer measurements alone. Although our study accounts for *p*CO_2_ variations in seasonality extremes, it does not account for variations due to diurnal cycles and episodic events such as flooding. However, the diurnal effect on CO_2_ emissions from estuarine surface waters is likely minimal, with differences between day and night driven more by tidal influence^65–67^. Episodic events are more significant drivers of increased CO_2_ emissions in estuarine surface waters^68^, but quantifying the effect of these events on CO_2_ emissions in Australian estuaries was beyond the scope of this study.

The seasonal variability of water-air CO_2_ fluxes in Australian estuaries is consistent with other studies globally, showing a range of change between seasons. For example, mean CO_2_ water-air fluxes were highest in autumn (36.2 mmol C m^−2^ d^−1^) (mean water temperature: 11.5 °C) followed by spring (24.1 mmol C m^−2^ d^−1^) (7.5 °C) and summer (18.2 mmol C m^−2^ d^−1^) (17.5 °C), and lowest in winter (7.9 mmol C m^−2^ d^−1^) (2.9 °C) in the temperate Tay estuary (tidal system) in the United Kindom^69^. In contrast, significantly higher mean water-air CO_2_ fluxes were found in winter (15.6 ± 5.2 mmol C m^−2^ d^−1^) and summer (13.4 ± 22.2 mmol C m^−2^ d^−1^, highest discharge rate) than in spring (−13.7 ± 16.4 mmol C m^−2^ d^−1^) and autumn (2.7 ± 6.6 mmol C m^−2^ d^−1^) in the Delaware Estuary, USA (tidal system) (2015 water temperature range: 0.4 °C to 28.6 °C^70^, temperature data: https://waterdata.usgs.gov/monitoring-location/01463500/#parameterCode=00010&startDT=2015-01-01&endDT=2016-01-01). In the current study, there was no correlation between water-air CO_2_ fluxes and temperature across all estuaries and within estuary types (studied estuary temperature range: 16 °C to 34.3 °C), except for a weak, significant correlation in tidal systems. This suggests that water-air CO_2_ fluxes in our study were likely driven by factors other than temperature, for example, riverine and lateral inputs into the estuaries or residence times.

Importantly, the seasonal variability between summer and winter water-air CO_2_ flux rates was small compared to the variability within individual estuaries and estuary types. Variability within the estuary types ranged from a maximum per-minute water-air CO_2_ flux rate 10 times (9 times as an estuary average minimum) larger than the minimum rate in the lagoons, 156 (113) times larger in the small deltas, and 165 (13) times larger in the tidal systems (Table 1). This larger within-estuary type spatial variability in water-air CO_2_ flux rates was captured in our continuous sampling along each estuary. We also accounted for the likely range of seasonal variability in water-air CO_2_ flux rates (maximum in summer, minimum in winter) by applying a summer:winter ratio to our summer data. As such, we argue that our annual emissions estimates are fairly robust.

Lagoons had the strongest disturbance signal, with *p*CO_2_ and water-air CO_2_ fluxes increasing with increasing disturbance (Figure 3A1 and B1), mainly driven by changes in the extent of seagrass cover. With increasing disturbance, NSW lagoons had a general decrease in seagrass cover (low = 55%, moderate = 19%, high = 24%, and very high = 3%)^47^ and mean dissolved oxygen (low = 123%_sat_, moderate = 97%_sat_, high = 95%_sat_, and very high = 108%_sat_) (Supplementary Fig. 4C2). Despite relying on data that was over 15 years old (mapped in 2007–2009)^71,72^, percent seagrass cover had a strong, negative association with *p*CO_2_ and a weaker, negative association with water-air CO_2_ fluxes (Supplementary Fig. 5 and Supplementary Results). These relationships suggest that higher *p*CO_2_ and water-air CO_2_ fluxes reflect decreased CO_2_ uptake by benthic vegetation (i.e. a less autotrophic system), as reported in several seagrass studies (e.g. refs. ^43,44,73^). High DOC concentrations in the low-disturbance lagoons were also consistent with DOC release from benthic vegetation, as observed in previous studies^48,74,75^ (Supplementary Fig. 4A2, Supplementary Table 5, and Supplementary Results). High percent O_2_ saturation in the very high disturbance lagoons (e.g. Curl Curl Lagoon; Supplementary Results, Supplementary Data 2, and Supplementary Table 4) was most likely due to a switch in production from benthic microalgae and macroalgae to phytoplankton^76^, enabling enhanced *p*CO_2_ drawdown and negative water-air CO_2_ flux rates (Figs. 3A1, B1).

**Fig. 4:**
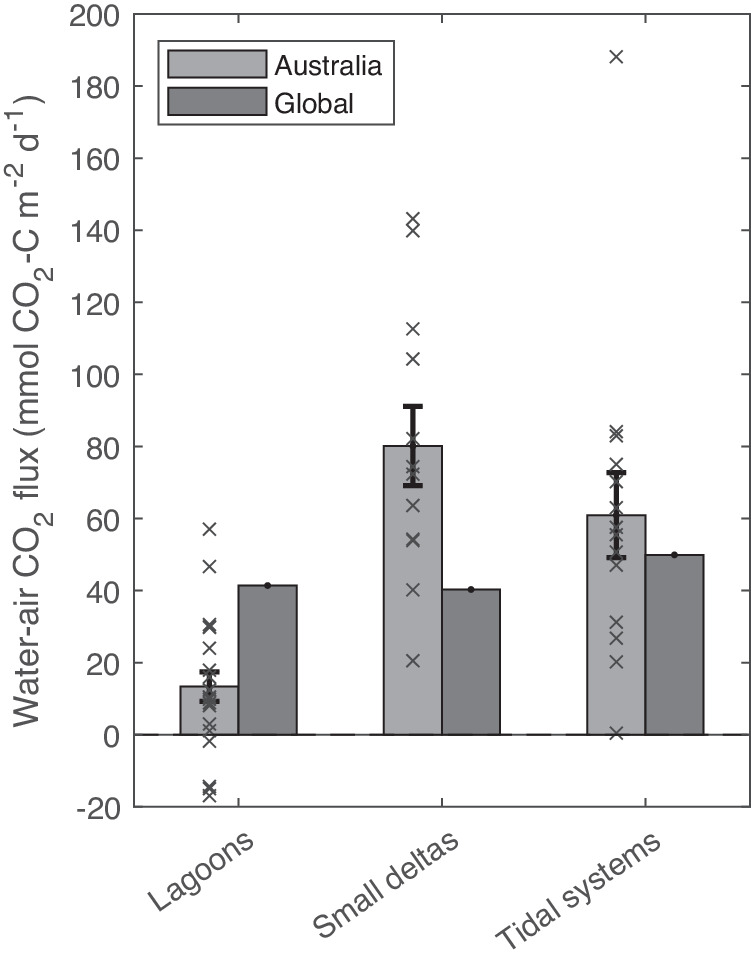
Australian and global estuarine CO_2_ emissions by estuary type. Mean water-air CO_2_ fluxes in Australian estuaries (*n*: Lagoons = 21, Small deltas = 12, and Tidal systems = 14) and in global estuaries^10^ from the three estuary types defined in this study. Error bars represent standard errors. Source data are provided as a Source Data file.

This study estimates that Australia’s estuaries have a mean (±SE) annual area-weighted water-air CO_2_ emissions of 44.6 ± 6.4 mmol CO_2_-C m^−2^ d^−1^, which is 25% and 110% greater, respectively, than estimates of global means of 35.6 mmol CO_2_-C m^−2^ d^−1^ ^10^ and 21.2 mmol CO_2_-C m^−2^ d^−1^^9^. However, the role of estuary type in CO_2_ flux rates had a significant impact on our estimates of Australian water-air CO_2_ flux. Annual mean (±SE) area-weighted water-air CO_2_ fluxes of the lagoons upscaled to all Australian lagoons (13.4 ± 4.1 mmol CO_2_-C m^−2^ d^−1^) was 68% lower than from global lagoon estimates (41.4 mmol CO_2_-C m^−2^ d^−1^)^10^. In contrast, annual mean water-air CO_2_ fluxes (Table 2 and Fig. 4) scaled to all Australian small deltas (80.1 ± 11 mmol CO_2_-C m^−2^ d^−1^) and tidal systems (60.9 ± 11.8 mmol CO_2_-C m^−2^ d^−1^) were 99% and 22% higher, respectively, than from global small deltas (40.3 mmol CO_2_-C m^−2^ d^−1^) and tidal systems (49.9 mmol CO_2_-C m^−2^ d^−1^)^10^. In addition to the higher water-air CO_2_ fluxes in Australian small deltas and tidal systems, the lower global mean water-air CO_2_ flux for all estuaries combined compared to Australia likely also reflects the contribution of fjords and fjards to global estimates^9,10^. Globally, fjords and fjards have been shown to take up CO_2_ from the atmosphere (median 66 Tg CO_2_ yr^−1^)^11^, but are absent in Australia.

Lower mean CO_2_ emissions in Australian lagoons compared to lagoons globally are likely due to overall lower disturbance in Australia. In addition, it may also reflect the greater abundance of ICOLLs in Australia (21% of global occurrence^41,77,78^). Isolation from marine waters, low riverine flow, and long residence times in ICOLLs may enhance autotrophic drawdown of CO_2_ by abundant seagrasses, resulting in smaller water-air CO_2_ fluxes (Table 2) than observed in non-Australian lagoons^43,79,80^. The weak hydrological connection between ICOLLs and rivers would also limit the input of CO_2_ supersaturated river water.

Higher mean water-air CO_2_ fluxes in Australian subtropical and tropical small deltas and tidal systems, compared to small deltas and tidal systems globally was an unexpected finding. Subtropical and tropical estuaries have previously been estimated to have lower water-air CO_2_ fluxes than systems at temperate latitudes^9^. We were unable to explicitly test for climate as we do not have sufficient estuaries of each geomorphic type and disturbance in each climate zone. However, macrotidal northern Australian small deltas and tidal systems are different from the small deltas and tidal systems in previous studies^81^ (Fig. 6 in Matthews and Matthews^82^) as North Australian tidal systems are dominated by extensive mangrove cover and have significantly greater tidal ranges (>4 m). Larger tidal ranges would lead to greater lateral inorganic and organic carbon export from mangroves to the tidal systems^56,58,83^. Higher mean water-air CO_2_ fluxes may also reflect the longer residence times resulting from characteristically low Australian freshwater inflows^84,85^. Long residence times would allow more time for CO_2_ produced from lateral inputs of DIC, DOC, and POC, and DIC from increased DOC and POC decomposition to be emitted across the water-air interface rather than flushed to the ocean.

Collectively, estuary geomorphic type is more important than disturbance in Australia, resulting in higher mean CO_2_ emissions from Australian estuaries despite their lower overall disturbance compared to global estuaries. The climate zone also has an important control on estuarine geomorphic type (e.g. tropical and subtropical mangrove-dominated macrotidal estuaries). This study suggests that relative to their surface area, Australian estuaries contribute a disproportionately large amount of CO_2_ emissions annually to global estuarine emissions. Using surface area estimates for Australian (62,100 km^2^; calculated from Table 3 in Chen et al.^9^) and global estuaries (1,012,440 km^2^) and global estuarine CO_2_ emission estimates by Laruelle et al.^10^ and Chen et al.^9^, Australian estuaries emit a mean (±SE) of 12.1 ± 1.7 Tg CO_2_-C annually. These emissions account for 12% or 8% of the estimated mean global estuarine CO_2_ emissions of 0.1 Pg CO_2_-C yr^−1^^9^ or 0.15 Pg CO_2_-C yr^−1^^10^, despite Australian estuaries accounting for only 6.1% of their calculated global estuarine surface area. This estimate includes the surface area coverage of the estuary types and disturbance groups and is dependent on the accuracy of surface areas for Australian and global estuaries. For instance, total estuarine water area of 39,390 km^2^ has been reported for Australia (Table 3)^40^, which is 57% greater than estimated by Dürr et al.^2^ (25,056 km^2^) and 37% smaller than estimated by Laruelle et al.^10^ (62,100 km^2^). Applying the estuarine surface areas of Australia’s National Land and Water Resources Audit (NLWRA)^40^ to data collected in the current study, Australian estuaries are estimated to emit (mean ± SE) 8.67 ± 0.54 Tg CO_2_-C annually (Table 2).

Australian tidal systems contributed the majority of the mean (±SE) annual CO_2_ emissions (8.18 ± 0.91 Tg CO_2_-C yr^−1^, 94.4%), with far smaller contributions from lagoons and small deltas (Table 2). Although lagoons in Australia (8.6% of total area) cover six times the estuarine surface area of small deltas (1.5% of total area;), CO_2_ emissions from lagoons were disproportionately low (0.27 ± 0.05 Tg CO_2_-C yr^−1^, 3.1% of Australian estuarine emissions) compared to small deltas (0.21 ± 0.03 Tg CO_2_-C yr^−1^, 2.5%) reflecting smaller water-air fluxes in lagoons (Table 2). The proportions of CO_2_ emitted by the different geomorphic types of estuaries in Australia were different from the proportions reported globally. For example, lagoons globally account for a larger proportion of CO_2_ emissions (31%; 0.046 Pg CO_2_-C yr^−1^) than small deltas (13%; 0.019 Pg CO_2_-C yr^−1^), and tidal systems only contribute 41% of global emissions (0.063 Pg CO_2_-C yr^−1^)^10^. The remaining proportion is made up of limited or non-filtering estuary types such as large rivers, karst-dominated coasts, and arheic coasts^2^. Furthermore, global CO_2_ emission estimates incorporate the contribution of fjords and fjards, which have the lowest water-air CO_2_ flux or show CO_2_ uptake but account for close to half of the global estuarine surface area^10,11^. Therefore, differences in Australian and global estuarine CO_2_ emissions are driven mostly by the geomorphic type (related to the climate zone of the estuary). This highlights the need to include geomorphic types in global CO_2_ emission assessments.

Geomorphology and disturbance influence water-air CO_2_ fluxes in Australian estuaries as a result of decreased hydrological connectivity in lagoons, and increased upstream riverine lateral inputs and tidal influence in small deltas and tidal systems. Water-air CO_2_ flux rates increase with higher disturbance, but geomorphology and disturbance interact, with the strongest disturbance signal in the lagoons, and a weak disturbance signal in the small deltas and tidal systems. Seasonal variations in CO_2_ emissions were a less important control on water-air CO_2_ fluxes in Australian estuaries. Previous global estuarine CO_2_ emission estimates have included geomorphology^9–11^, but not disturbance or the two factors together. CO_2_ emissions for global lagoons could therefore be over-estimated due to the bias towards more disturbed systems in the northern hemisphere. In contrast, CO_2_ emissions for global small deltas and tidal systems could be under-estimated due to the bias towards temperate systems in the northern hemisphere. As such, upscaling of global estuarine CO_2_ emissions should be based on geomorphic estuary-types but also consider land-use disturbance and climate and ideally, their interaction with geomorphic type.

## Methods

In 36 estuaries around Australia, *p*CO_2_, DIC, DOC, physicochemistry, and physical parameters (wind speed, depth, current velocity, and barometric pressure) were measured along the salinity gradient from the marine to freshwater endmember (where possible). Data were combined with published CO_2_ fluxes and water quality data for 11 other Australian estuaries^12,30^, giving a total of 47 Australian estuaries. The same survey methods were used in all 47 estuaries. *p*CO_2_ and water-air CO_2_ fluxes were classified according to estuary type (lagoons, small deltas, and tidal systems) and disturbance group (low, moderate, high, and very high) and analysed for significant differences. Finally, the classified water-air CO_2_ fluxes were upscaled to all of Australia and mean estimates were compared to previous global mean estimates of estuary CO_2_ emissions. While we provide a full set of statistics in this study, we use the mean (±SE) for global comparison because our high-resolution water-air CO_2_ fluxes over a range of disturbance classes and geomorphic estuary types was better represented by the means than medians.

### Estuary classification schemes

Estuaries were selected to cover a large range of disturbance and geomorphic types according to the classifications of NLWRA^40^ and Dürr et al.^2^. NLWRA^40^ assessed 971 Australian estuaries and described four disturbance classes (low (near-pristine), moderate (relatively unmodified), high (modified), and very high (extensively modified)). These disturbance groups were qualitatively classified based on changes in catchment land-use, estuary use, and ecology (Supplementary Table 6) and provided an assessment that was more relevant than adopting a single set of indicators. This is because the Australian continent covers a large surface area, encompassing over 1000 estuaries and climatic variations, making a single set of disturbance indicators likely misleading^86,87^. The global estuarine typology of Dürr et al.^2^ details three geomorphic types found in Australia: (1) lagoons (including Intermittently Closed or Open Lakes and Lagoons (ICOLLs) and estuaries with a central basin morphology), (2) small deltas, and (3) tidal systems (including drowned river valleys and tidal embayments), based around morphological and sedimentation characteristics driven by tidal influence (classification criteria in Supplementary Table 7). However, the existing classification of Australian estuaries^2^ did not match our observations of satellite imagery, because it was developed with a low spatial resolution (0.5°, or 50 km). Therefore, all 971 Australian estuaries were re-classified into the three estuary types by distinguishing physical characteristics based on the criteria of Dürr et al.^2^ (Supplementary Table 7) using satellite imagery (Google Earth) (Supplementary Data 3). Water surface area for 108 estuaries with missing surface area measurements were also calculated using satellite imagery (Google Earth). The re-classified estuary database was then combined with the estuarine disturbance database in NLWRA^40^ (dataset URL: https://data.gov.au/dataset/ds-aodn-8fec03d6-48e3-4352-9ddb-085e42e55637/details?q=, Supplementary Data 3).

**Fig. 5:**
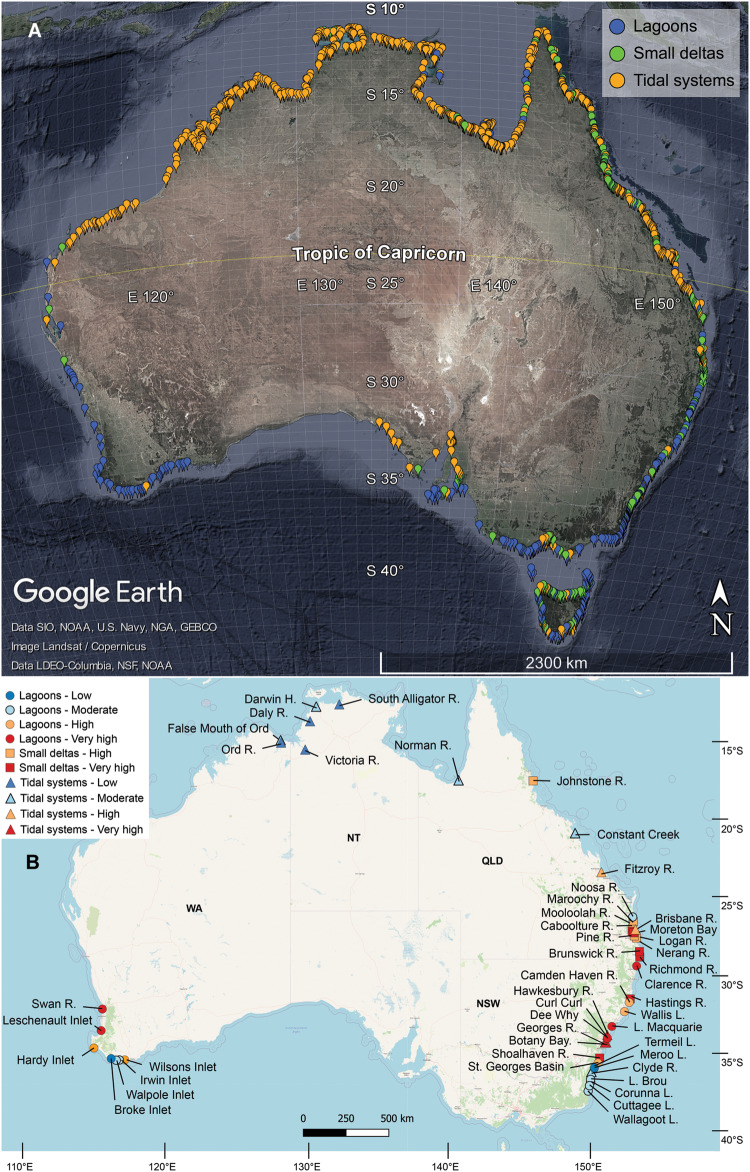
Distribution of estuary types and study locations in Australia. **A** Estuaries in Australia^40^ classified into three estuary types based on conceptual definitions (Supplementary Table 7) by Dürr et al.^2^ (©Google Earth) and the (**B**) location of study estuaries in Australia according to estuary type (shapes) and disturbance class (colours) (©OpenStreetMap, https://www.openstreetmap.org/copyright). H.: Harbour, R.: River, and L.: Lake.

The spatial distribution of estuary types in Australia corresponds to the tidal ranges of their respective coastlines (Fig. 5A). Tidal systems dominate the macro-tidal regions of northern Australia, whereas lagoons are found mostly in the micro-tidal regions of southern Australia. All three estuary types with all four disturbance groups, except for low and moderate disturbance in small deltas, were included in our estuary selection (Table 3). The surface area of estuaries sampled and included in this study represents 12.3% of the total Australian estuarine surface area, consisting of 19.1% of lagoons, 20.1% of small deltas, and 11.6% of tidal systems in Australia (Table 3).

### Study sites

Measurements from the 36 estuary surveys and from the 11 published estuary surveys were taken over the austral spring-summer season (Fig. 5B and Supplementary Data 1). The estuaries included in this study were comprised of 21 estuaries in New South Wales (Nov to Dec 2017), one in southeast Queensland (Moreton Bay, Oct 2018), seven along the north Australian coastline (from Karumba, Queensland to Wyndham, Western Australia, Oct to Dec 2018), seven along the south-west coastline of Western Australia (from Albany to Perth, Feb to Mar 2019), three in north-east Queensland^30^ (late spring Sept to Oct 2014) and eight in southeast Queensland^12^ (late spring Oct 2016) (Fig. 5B and Supplementary Data 1). Percent seagrass coverage for the New South Wales estuaries was obtained from Roper et al.^47^. Termeil Lake and Lake Brou were excluded from seagrass coverage analysis because although zero coverage was recorded by Roper et al.^47^, extensive seagrass cover was observed during our surveys.

### Underway data measurements

Using a 6 m research vessel, physicochemical parameters and *p*CO_2_ were measured along a transect encompassing the length of each of the 36 estuaries, starting at the river mouth just after high-tide and ending in freshwater (salinity ~2). Although we aimed to finish the surveys at salinity ~2, this was not always possible because of shallow water and/or natural and artificial obstacles. As such, estuary data in this study reflect the spatial variations along the estuarine gradient (marine to upstream-riverine). A cruising ground speed of ~8 km h^−1^ was maintained where possible to ensure spatial and temporal consistency. The surveys were carried out during daylight hours, typically lasting over the course of a day. Surveys in large estuaries often required 2 to 3 days but never exceeded five days (Supplementary Data 1). *p*CO_2_ was recorded at one-minute intervals using an integrated water-gas loop setup (Supplementary Fig. 6).

Water was continuously pumped from beneath the hull (0.5 m to 1 m water depth) at ~1800 l^−1^ h^−1^ using a 12 V pump with backflow prevention (800GPH, Rule) to a high-flow filter basket (Ozito) before entering a two-way split. One path led to a flow-through chamber with a multi-parameter sonde (HL4, Hydrolab) measuring salinity (±0.5%), temperature (±0.1 °C), pH_NBS_ (±0.2), and dissolved oxygen (DO_%sat_; ±2%). The second path entered a loop consisting of a pair of interconnected showerhead exchangers (RAD Aqua, Durridge) equilibrating dissolved gases in the water with the headspace. The dried gas stream was then measured for CO_2_ concentration, using a LiCOR 840 A CO_2_ gas analyser (accuracy <1%) and a Picarro G-2508 Cavity Ring-Down Spectrometer (CRDS, ±0.05%)^87^. In the ICOLL lagoons (indicated in Supplementary Data 1) where a smaller boat was used, CO_2_ measurements were only taken with the LiCOR 840 A CO_2_ gas analyser. Measured CO_2_ was humidity-corrected and in-situ *p*CO_2_ was calculated using methods in Pierrot et al.^88^. The LiCOR 840 A was calibrated using a two-step process with low (250 ppm) and high (8000 ppm) *p*CO_2_ gas standards. The CRDS was serviced and calibrated by the manufacturer (Picarro, USA) before each field trip.

### Discrete water samples, morphological, and meteorological data

Water samples were collected for DIC and DOC concentrations, along with estuarine (depth and water current velocity) and meteorological measurements at the start and end of survey transects and at salinity intervals of ~5. In cases where salinity did not change much (<5) along the survey, samples were collected every hour instead (i.e. every 8 km of estuary travelled). In the ICOLL lagoons (as indicated in Supplementary Data 1) where no significant salinity gradient was present, discrete water samples were collected from at least 3 points across the estuary. For DOC, 30 ml water samples were filtered through a pre-combusted (500 °C, ~5 h) 0.7 µm GF/F filter (Whatman, Merck) into an acid-washed glass vial containing 100 µl of 85% phosphoric acid (H_3_PO_4_). 50 ml water samples for DIC analysis were syringe-filtered (0.45 µm SFCA Minisart, Sartorius) into a crimp-top glass bottle without any headspace and preserved with 30 µl mercuric chloride (HgCl_2_). DOC concentrations were determined using a total organic carbon analyser (±3%; 1030 W, Aurora)^89^. DIC concentrations were analysed with a Marianda AIRICA coupled to a CO_2_/H_2_O analyser (LI7000, LiCOR), calibrated for accuracy with certified reference material^90^ at a typical precision of better than 2 µmol kg^−1^ ^91^. All samples were processed immediately, stored on ice while the survey was underway, and frozen (−20 °C) as soon as possible (typically within five hours of collection) except for DIC, which was stored at room temperature. On the main research vessel and the smaller boat, water current velocity was measured using a differential GPS-assisted Lagrangian method with a neutrally-buoyant drifter (adapted from Wetzel and Likens^92^). Current velocity measurements likely indicated flow rates of the ebbing tide, as the surveys were carried out after the turn of the high tide. Water depth on the main research boat was measured using a hull-mounted acoustic transducer (Airmar), while water depth was measured using a lead and line on the smaller boat or taken from Roper et al.^47^. Barometric pressure (±0.5hPa @20 °C), air temperature (±1.1 °C @20 °C), and true wind speed (±5% @10 m s^−1^) were measured 3 m above the water surface using a vessel-mounted weather station (200WX, Airmar). In the ICOLL lagoons, daily averaged meteorological data were obtained from the closest Bureau of Meteorology (BOM) weather station (Climate Data Online^93^).

### Water-air CO_2_ flux calculations

Water-air CO_2_ flux (*F*CO_2_; mmol CO_2_-C m^−2^ d^−1^) was calculated at 1-minute intervals using Eq. (1):

1 $$F={k}_{600}{K}_{0}({C}_{{water}}-{C}_{{air}})$$

where *k*_600_ is the gas transfer velocity (m d^−1^), *K*_0_ is the solubility coefficient of CO_2_ (mol l^−1^ atm^−1^), and *C*_*water*_ and *C*_*air*_ are the partial pressure of CO_2_ (µatm) in water and air, respectively^94^. The formula from Weiss^95^ was used to obtain CO_2_ solubility coefficients based on salinity and temperature Eq. (2):

2 $${ln}\,{K}_{0}={A}_{1}+{A}_{2}\left(\frac{100}{T}\right)+{A}_{3}{ln}\left(\frac{T}{100}\right)+S\left[{B}_{1}+{B}_{2}\left(\frac{T}{100}\right)+{B}_{3}{\left(\frac{T}{100}\right)}^{2}\right]$$

where *K*_0_ is expressed in moles L^−1^ atm^−1^, *A*_1_ (-58.0931), *A*_2_ (90.5069), *A*_3_ (22.2940), *B*_1_ (0.027766), *B*_2_ (-0.025888), and *B*_3_ (0.0050578) are constants, *T* is absolute temperature, and *S*‰ is the salinity. CO_2_ atmospheric concentration was assumed to be 407 µatm^96^, which was the mean concentration in 2018. Although *k*_600_ is a significant variable required for calculating water-air fluxes, measuring *k*_600_ in-situ was not feasible due to the large spatial coverage of this study. As such, five empirical *k*_600_-models for a range of coastal-marine ecosystems were used from the literature to estimate mean *k*_600_ (equations (6) to (10) listed in Table 4), i.e., mangrove-dominated^28,97^ and tidal^27^ (using wind speed, water depth, and current velocity), lagoonal^53^ (using wind speed and water depth), and marine-dominated^94^ (using wind speed only) coastal ecosystems. Windspeed is corrected for a height of 10 m (*U*_10_) by rearranging the formula from Amorocho and DeVries^98^:

3 $${U}_{z}={U}_{10}\left[1-\frac{{\left({C}_{10}\right)}^{\frac{1}{2}}}{k}{{{{{\rm{ln}}}}}}\left(\frac{10}{z}\right)\right]$$

where *U*_*z*_ is the measured windspeed at *z* height (3 m) in m s^−1^, *C*_10_ is the surface drag coefficient for wind at 10 m (1.3 × 10^−3^)^97^, and *κ* is the Von Karman constant (0.41). In the first four parameterisations (Eqs. (6) to (9) in Table 4), *k*_600_ is the gas transfer velocity (cm h^−1^) normalised to a Schmidt number of 600. The parameterisation in equation 10 (Table 4) by Wanninkhof^94^ calculated *k* at the Schmidt number (Sc) of the measured temperature and salinity, converted to *k*_600_ using Eq. (4):

4 $${k}_{600}=k{\left(\frac{600}{{Sc}}\right)}^{-0.5}$$

**Table 4:** Gas transfer velocity normalised to Schmidt number of 600 (*k*_600_) parameterisations using various methods in published literature

Literature	*k*_600_ parameterisations	Method	Study area	Eqn.
Rosentreter et al.^97^	*k*_600_ = −0.08 + 0.26 *v* + 0.83*U*_*10*_ + 0.59 *h*	Flux chamber	Three mangrove estuaries	6
Borges et al.^27^	*k*_600_ = 1 + 1.719*v*^0.5^ *h*^−0.5^ + 2.58*U*_*10*_	Flux chamber	Macrotidal estuary (Scheldt)	7
Jiang et al.^53^	*k*_600_ = 0.314 *U*_*10*_^2^ - 0.436*U*_*10*_ + 3.99	Predictive modelling	Global	8
Ho et al.^28^.	*k*_600_ = 0.77 *v*^0.5^ *h*^−0.5^ + 0.266*U*_*10*_	^3^He/SF_6_	Mangrove estuaries	9
Wanninkhof^94^	*k* = 0.251*U*_*10*_^2^ (*Sc*/660)^−0.5^	Global ocean inverse model	Global	10

*v* is the current velocity in cm s^−1^, *h* is the water depth (m), and *U*_10_ is the wind speed (m s^−1^) at 10 m height calculated according to Amorocho and DeVries^98^.

A Schmidt exponent of -0.5 was used to account for higher water turbulences associated with tidal currents in estuaries^99^. To calculate water-air CO_2_ fluxes, *k* was derived from *k*_600_, which was calculated using the other four parameterisations (Eqs. (6) to (9) in Table 4) by rearranging Eq. (4). *Sc* at the measured temperature and salinity was calculated using the formula in Wanninkhof^94^ (Eq. (5)):

5 $${Sc}=A+{Bt}+{{Ct}}^{2}+{{dt}}^{3}+{{Et}}^{4}$$

where *A*, *B*, *C*, *D*, and *E* are constants for CO_2_ in fresh water (1923.6, −125.06, 4.3773, −0.085681, and 0.00070284, respectively) and seawater (2116.8, −136.25, 4.7353, −0.092307, 0.0007555, respectively), and *t* is temperature in °C. A salinity factor was calculated from the difference between freshwater and sea water *Sc* and applied to calculate *Sc* at the measured salinity.

To ensure consistency between water-air CO_2_ fluxes measured for estuaries in this study and those previously reported for eight southeast Queensland estuaries^12^, water-air CO_2_ fluxes were recalculated using the five parameterisations. Water-air CO_2_ fluxes from the previously reported three north Queensland estuaries^30^ were not recalculated because water depth and current velocity data were unavailable. However, given that these three estuaries were each categorised in a different disturbance group and/or estuary type (moderate and high disturbance tidal system, and a high disturbance small delta, Table 3), this should not introduce any systematic bias.

### Data processing and statistical analysis

Per-minute *p*CO_2_ and water-air CO_2_ flux were averaged to 5-minute datapoints to reduce the number of data points whilst maintaining the high resolution and main features of the dataset. Kolmogorov-Smirnov and Levene’s tests for normality and homoscedasticity, respectively, returned significant results (*p* < 0.05), ruling out parametric methods for statistical analysis. Consequently, significant differences (α = 0.05) between and within estuary types (3 factors) and disturbance groups (4 factors) were tested using the Permutational Multivariate Analysis of Variance (PERMANOVA) procedure with Euclidean distance as the dissimilarly matrix in Primer v7 and PERMANOVA+ add-on (PRIMER-e). The dataset for PERMANOVA analysis was normalised (*z*-score) but not power-transformed to retain the heterogeneity between the mean and variances, retaining the spatial scale along the estuarine transect. Not power-transforming the data reduces the possibility of an inflated type I error^100^. Salinity was included as a covariate in the *p*CO_2_, water-air CO_2_ flux, DOC and DIC analyses to remove differences influenced by salinity. The focus of PERMANOVA is on the differences between the data points rather than descriptive statistics (mean, median, etc.). PERMANOVA can therefore, identify significant differences in datasets even where there are similarities in the descriptive statistics. 9999 permutations were performed using residuals under a reduced model using type I sum of squares. Significant results were further analysed with pairwise PERMANOVA. *p*CO_2_ and water-air CO_2_ fluxes were analysed for correlations with salinity using Pearson’s correlation and combined with physicochemical data, DOC, DIC, and percent seagrass cover to analyse for partial correlations while controlling for the effect of salinity (α = 0.05) in SPSS v25 (IBM). Data used for correlation analysis were power-transformed where necessary and normalised (*z*-score). Partial correlation analysis was chosen over multivariate methods such as Principal Component Analysis (PCA) because targeted testing for correlations between variables and CO_2_ was more useful than an exploratory approach.

### CO_2_ emission upscaling to the Australian continent

Published summer and winter water-air CO_2_ flux rates were available for 13 Australian estuaries, including each of the three geomorphic estuary types (Supplementary Table 1). Of these, 10 of the estuaries were included in this study^12,30^, along with an additional three from a published study^43^. The summer and winter water-air CO_2_ fluxes were used to calculate a summer:winter water-air CO_2_ flux ratio (mean and range) for each of the three estuary types (Supplementary Table 2). Ratios for each estuary type were then applied to the measured summer water-air CO_2_ fluxes for the 47 estuaries to estimate the mean and range of winter water-air CO_2_ fluxes (Table 2). The summer and winter mean water-air CO_2_ flux rates from each estuary were averaged together to derive the annual water-air CO_2_ flux rates and emissions for the 47 estuaries. To gauge the sensitivity of annual Australian emissions to winter flux rates, the summer mean flux rates were also adjusted up and down by the minimum and maximum of winter:summer ratios (Supplementary Table 2). Upscaled fluxes determined based on these minimum and maximum ratios allow an upper and lower limit to be placed on these estimates.

Annual CO_2_ emissions from the 47 estuaries were upscaled to all Australian estuaries (*n* = 971) by multiplying the estuary type-specific and disturbance-specific water-air CO_2_ fluxes (mmol CO_2_-C m^−2^ d^−1^) by the total estuarine surface area of the relevant systems^40^ (Table 3). Small deltas with low to moderate disturbance were not available for this study. However, even though measured low and moderately disturbed small delta water-air CO_2_ fluxes were likely different from the mean high and very high disturbed small delta water-air CO_2_ flux, their impact on total Australian estuary emissions is low. This is because small deltas only make up 1.5% of Australia’s estuarine surface area (Table 3).

### Reporting summary

Further information on research design is available in the Nature Portfolio Reporting Summary linked to this article.

### Supplementary information

Supplementary Information

Peer Review File

Description of Additional Supplementary Files

Supplementary Data 1

Supplementary Data 2

Supplementary Data 3

Reporting Summary

### Source data

Source Data

## Data Availability

The environmental survey data generated/used in this study is freely available and has been deposited in the FigShare database under accession code 10.6084/m9.figshare.25242676. Figure source data are provided with this paper as part of the Supplementary Information. Source data are provided with this paper.
