# Supplementary material for: High carbon dioxide emissions from Australian estuaries driven by geomorphology and climate
Source: Nat Commun. 2024 May 10;15:3967. doi: 10.1038/s41467-024-48178-4 (PMC11087516; doi:10.1038/s41467-024-48178-4)
Supplement: Supplementary file 1 — Supplementary Information [file 41467_2024_48178_MOESM1_ESM.pdf]

High carbon dioxide emissions from Australian estuaries driven by geomorphology and climate

SUPPLEMENTARY INFORMATION

Results - Physicochemistry, dissolved carbon concentrations, and seagrass cover

Mean salinity was highest in tidal systems (28.1, *n*=1138), followed by lagoons (25.8, *n*=751), and lowest in small deltas (23.1, *n*=719) (all *p*=0.001) (Supplementary Figure 3A and Supplementary Table 3). Increasing mean salinity significantly corresponded to higher disturbance in lagoons (*n*: low=41 and high=261) (all *p*=0.001 with *p*=0.049 in high to very high groups) except between moderate (*n*=161) and very high (*n*=288) disturbance lagoons (*p*=0.383) (Supplementary Figure 3BSupplementary Figure and Supplementary Table 3). Mean salinity significantly decreased from highly to very highly disturbed small deltas (*p*=0.007) (*n*=353 and 366, respectively, Supplementary Figure 3C) and tidal systems (*p*=0.001) (*n*=117 and 234, respectively) but significantly increased from moderately to highly disturbed tidal systems (*p*=0.005) (Supplementary Figure 3D and Supplementary Table 3). There was no significant difference between low (*n*=315) and moderate disturbance (*n*=472) tidal systems (*p*=0.278) (Supplementary Figure 3D and Supplementary Table 3). Increasing salinity was inversely correlated with pCO_2 and water-air CO_2 fluxes in small deltas and tidal systems (Pearson's correlation, small deltas pCO_2: *r*=-0.541, *p*=0.001 and CO_2 flux: *r*=-0.361, *p*=0.001; tidal systems pCO_2: *r*=-0.565, *p*=0.001 and CO_2 flux: *r*=-0.373, *p*=0.001), but there was no significant correlation in lagoons (pCO_2: *p*=0.441 and CO_2 flux: *p*=0.465). To exclude the effect of salinity on CO_2, partial correlations were used to test the influence of disturbance within estuary types with salinity as a covariate. DOC and DIC concentrations differed significantly between the estuary types. DOC concentrations were highest in lagoons (*n*=104) and lowest in tidal systems (*n*=103) (Supplementary Figure 4A1 and Supplementary Table 5), whereas DIC concentrations were significantly higher in the tidal systems (*n*=103) and lowest in the lagoons (*n*=100, small deltas: n_{DIC}=149, n_{DOC}=150) ($p \leq 0.005$) (Supplementary Figure 4B1 and Supplementary Table 5). Across all estuaries, there was no significant effect of disturbance on DIC concentrations (*p*=0.735, whereas DOC differed significantly: *p*=0.002) but disturbance affected DOC and DIC concentrations within

the estuary types (p=0.001) (Supplementary Figure 4A2 to 4A4 and 4B2 to 4B4 and Supplementary Table 5). This suggests that estuary type influences the effects of disturbance in estuaries. Increasing tidal range significantly correlated to higher pCO_2 (r=0.298, p=0.001) and water-air CO_2 flux (r=0.235, p=0.001) across all estuaries.

In lagoons, DOC concentrations were significantly lower with increasing disturbance in low to high disturbance systems ($p \leq 0.041$) but DOC concentrations were similar in high and very high disturbance systems ($p \geq 0.575$) (Supplementary Figure 4A2 and Supplementary Table 5). DIC concentrations were significantly lower with increasing disturbance ($p \leq 0.048$) but were similar between the moderate and high disturbance systems ($p \geq 0.528$) (Supplementary Figure 4B2 and Supplementary Table 5). In tidal systems, DOC concentrations were higher with increased disturbance ($p \leq 0.001$) but DOC concentrations were similar between the low and moderate disturbance systems, and moderate and high disturbance systems ($p \geq 0.142$) (Supplementary Figure 4A4 and Supplementary Table 5). DIC concentrations significantly decreased from the low to very high disturbance systems ($p \leq 0.041$), except for insignificantly higher DIC concentrations in the high disturbance system compared to low and moderate disturbance systems ($p \geq 0.594$) (Supplementary Figure 4B4 and Supplementary Table 5). In small deltas, DOC (p=0.746) and DIC (p=0.393) concentrations were not significantly different with increased disturbance (Supplementary Figure 4A3 and B3 and Supplementary Table 5).

Dissolved oxygen differed significantly between the estuary types ($p \leq 0.003$), with the highest concentrations in lagoons and the lowest in small deltas (Supplementary Figure 4C1 and Supplementary Table 4). In lagoons, dissolved oxygen was similar between the moderate and high (p=0.539), the low and very high ($p \geq 0.175$), and high and very high ($p \leq 0.05$) disturbance systems and significantly different between the other systems ($p \leq 0.035$) (Supplementary Figure 4C2 and

Supplementary Table 4). In tidal systems, dissolved oxygen in the high and very high disturbance systems was significantly lower compared to the moderate disturbance systems (p=0.001) but not significant in the other groups ($p \geq 0.184$) (Supplementary Figure 4C4 and Supplementary Table 4). In small deltas, dissolved oxygen was similar across disturbance groups (p=0.072) (Supplementary Figure 4C3 and Supplementary Table 4).

In all estuary types, dissolved oxygen was negatively associated with pCO_2 (lagoons: r=-0.516, p=0.001; small deltas: r=-0.372, p=0.001; tidal systems: r=-0.661, p=0.001) and water-air CO_2 flux (lagoons: r=-0.308, p=0.001; small deltas: r=-0.291, p=0.001; tidal systems: r=-0.528, p=0.001). In small deltas, DOC had a weak positive relationship with pCO_2 (r=0.312, p=0.001) and water-air CO_2 flux (r=0.318, p=0.001). DIC was weakly inversely associated with water-air CO_2 flux (r=0.177, p=0.031). In lagoons, DIC was positively associated (p<0.001) with pCO_2 (r=0.635) and water-air CO_2 flux (r=0.415). In NSW lagoons, percent seagrass cover was inversely associated with pCO_2 (r=-0.6, p=0.001) and water-air CO_2 fluxes (r=-0.352, p=0.018 (Supplementary Figure 5).

Supplementary Tables

Supplementary Table 1. Winter:summer ratios (seasonal ratios) calculated from published winter and summer water-air CO_2 flux rates in 13 Australian estuaries, representing each of the estuary types.

					Water-air CO_2 flux	
Literature	Estuary	Season	Estuary type	Disturbance	Mean (mmol CO_2 m^{-2} d^{-1})	Seasonal ratio
Maher & Eyre	Camden Haven River	Winter	Lagoon	High	-3.1	0.69
		Summer			-4.5	
Maher & Eyre	Wallis Lake	Winter	Lagoon	High	-5.3	0.58
		Summer			-9.1	
Rosentreter et al.	Johnstone River	Winter	Small delta	High	110.4	2.75
		Summer			40.2	
Wells et al.	Caboolture River	Winter	Small delta	High	45.5	0.75
		Summer			60.4	
Wells et al.	Logan-Albert River	Winter	Small delta	High	109.4	2.50
		Summer			43.7	
Wells et al.	Nerang River	Winter	Small delta	High	27.8	0.33
		Summer			84.3	
Wells et al.	Mooloolah River	Winter	Small delta	High	18.5	0.57
		Summer			32.8	
Wells et al.	Maroochy River	Winter	Small delta	High	94.5	0.82
		Summer			115.8	
Maher & Eyre	Hastings River	Winter	Small delta	Very high	3.3	4.71
		Summer			0.7	
Wells et al.	Pine River	Winter	Small delta	Very high	61.2	0.54
		Summer			113.1	
Wells et al.	Brisbane River	Winter	Small delta	Very high	42.8	0.47
		Summer			90.9	
Rosentreter et al.	Constant Creek	Winter	Tidal system	Moderate	21.6	0.43
		Summer			50.5	
Rosentreter et al.	Fitzroy River	Winter	Tidal system	High	95.9	2.17
		Summer			44.1	

Supplementary Table 2. Mean, minimum, and maximum seasonal ratios of each estuary type and across Australian estuaries.

	Seasonal ratio		
Estuary type	Minimum	Mean	Maximum
Lagoons	0.58	0.64	0.69
Small deltas	0.33	1.49	4.71
Tidal systems	0.43	1.30	2.17
**Australia**	**0.33**	**1.33**	**4.71**

Supplementary Table 3. Descriptive statistics calculated for salinity and temperature using data at per-minute resolution in the estuary types (est. type; LA: lagoons, SD: small deltas, and TS: tidal systems), disturbance (dist.) groups A: low, B: moderate, C: high, and D: very high), and disturbance groups within estuary types. SE: standard error; IQR: interquartile range (3rd quartile-1st quartile). The mean of minimum and maximum values calculated for each estuary are presented in brackets.

		Salinity						Temperature (°C)					
Estuary type	Dist. group	Mean	Median	SE	IQR	Min	Max	Mean	Median	SE	IQR	Min	Max
LA		25.8	27.6	2.1	15.6	2 (19.5)	42.1 (29.1)	24.2	24.3	0.5	2.6	16.2 (22.3)	29.7 (26.5)
SD		23.1	24.7	3.2	20.4	2 (9)	38.2 (36.3)	23.5	23.2	0.6	1.9	16 (21)	28.2 (25.1)
TS		28.1	31.7	2.1	8.2	2.1 (19.3)	37.3 (33.2)	28.9	31.3	1.1	6.9	16.9 (26.4)	34.3 (29.6)
	A	26.8	31.4	3.4	14.7	2.1 (17.6)	36 (24)	31.2	31.7	0.9	1.4	22.8 (28.7)	34 (30.6)
	B	27.8	31.7	2.4	8.5	3.4 (20.8)	37.7 (31)	29.0	31.5	1.1	7.2	19.7 (24.9)	34.3 (27.6)
	C	26.5	29.1	2.6	12.6	2 (18.3)	38.7 (35.6)	23.5	23.3	0.6	2.9	16.2 (20.8)	29.2 (25.3)
	D	24.2	26.9	2.7	18.0	2 (11.9)	42.1 (33.9)	24.0	24.2	0.6	2.8	16 (21.4)	29.7 (26.5)
LA	A	17.0	15.6	4.7	13.1	5.3 (16.2)	29.8 (17.6)	25.4	25.7	1.0	3.4	22.8 (25.3)	28 (26.8)
	B	25.1	25.4	3.6	22.0	8.2 (22.2)	37.7 (28.1)	23.8	24.0	0.4	1.6	21.9 (22.8)	26.8 (25.3)
	C	27.6	29.1	3.5	7.8	4.9 (22.6)	38.7 (34)	23.7	24.3	1.2	2.9	16.2 (19.1)	29.2 (26)
	D	26.0	27.9	4.3	16.4	2 (15.6)	42.1 (31.9)	24.8	24.9	1.1	3.5	19.8 (23)	29.7 (28.1)
SD	A	24.3	26.4	4.4	19.6	2 (11.4)	38.2 (37)	23.7	23.2	0.8	2.2	20.5 (21.8)	28.2 (25.2)
	B	22.0	23.3	4.5	20.9	2 (6.7)	37.7 (35.6)	23.2	23.2	0.9	1.9	16 (20.2)	27.5 (25)
TS	A	28.1	31.6	4.1	8.7	2.1 (18.7)	36 (28.8)	32.0	31.9	0.4	1.4	30.6 (31.2)	34 (33.5)
	B	28.8	31.7	3.4	5.3	3.4 (18.8)	37.3 (35.1)	30.7	31.8	1.3	1.4	19.7 (27.9)	34.3 (30.9)
	C	30.9	33.0	3.2	7.7	21.6 (28.4)	35.5 (35)	22.3	22.1	0.7	0.5	21.2 (22.2)	24.6 (23.4)
	D	25.5	30.4	5.2	13.7	2.1 (14.8)	34.7 (34.6)	24.4	24.9	1.3	1.8	16.9 (20.5)	26.8 (26.2)

Supplementary Table 4. Descriptive statistics calculated for pH and dissolved oxygen (%sat.) using data at per-minute resolution in the estuary types (est. type; LA: lagoons, SD: small deltas, and TS: tidal systems), disturbance (dist.) groups A: low, B: moderate, C: high, and D: very high), and disturbance groups within estuary types. SE: standard error; IQR: interquartile range (3rd quartile-1st quartile). The mean of minimum and maximum values calculated for each estuary are presented in brackets.

		pH						Dissolved oxygen (%sat.)					
Estuary type	Dist. group	Mean	Median	SE	IQR	Min	Max	Mean	Median	SE	IQR	Min	Max
LA		8.1	8.1	0.1	0.5	5.2 (7.7)	10 (8.5)	102.0	98.1	5.3	14.6	1.2 (83)	313 (139)
SD		8.1	8.2	0.2	1.0	5 (7.2)	9.3 (8.5)	83.7	87.0	6.5	17.2	14.9 (64.7)	183 (108)
TS		7.9	8.0	0.1	0.3	6.8 (7.6)	8.5 (8.1)	95.3	96.3	2.4	9.9	0.8 (81.7)	123 (106)
	A	8.0	8.0	0.1	0.3	7.5 (8.1)	10 (8.6)	99.4	98.1	6.3	16.1	0.8 (84.8)	176 (125)
	B	8.0	8.0	0.1	0.2	6.9 (7.7)	9.8 (8.3)	97.5	97.8	2.5	8.0	1.2 (81.2)	162 (117)
	C	8.0	8.1	0.2	0.9	5.2 (7.3)	9.2 (8.4)	87.4	91.5	6.5	17.7	14.9 (65.6)	183 (113)
	D	7.9	7.9	0.1	0.5	5 (7.3)	9.3 (8.4)	96.5	93.0	5.5	13.3	51.6 (82.9)	313 (130)
LA	A	8.8	8.5	0.3	0.8	8.3 (8.5)	10 (9.3)	123.4	114.8	13.3	39.6	93.8 (105.4)	176 (151)
	B	8.4	8.3	0.2	0.7	6.9 (7.9)	9.8 (8.5)	96.6	95.5	5.2	16.1	1.2 (77.2)	162 (122)
	C	7.8	8.0	0.3	0.5	5.2 (6.9)	8.6 (8.3)	95.3	97.2	7.3	12.0	35.5 (66.1)	142 (119)
	D	8.0	8.1	0.1	0.5	7.5 (7.8)	8.6 (8.3)	107.9	98.7	12.6	15.6	51.6 (92.9)	313 (169)
SD	A	8.4	8.5	0.2	0.6	6.8 (7.7)	9.2 (8.6)	77.4	80.5	12.0	23.0	14.9 (56.6)	183 (113)
	B	7.8	7.7	0.2	0.5	5 (6.7)	9.3 (8.4)	89.5	89.6	3.9	12.2	58.6 (72.8)	112 (103)
TS	A	7.9	8.0	0.1	0.3	7.5 (7.8)	8.2 (8.1)	96.2	97.6	6.1	15.1	0.8 (69.4)	123 (106)
	B	7.9	8.0	0.1	0.1	7.1 (7.5)	8.3 (8)	97.8	97.9	2.6	5.7	78.2 (86.7)	122 (109)
	C	7.3	7.1	0.3	0.3	6.8 (7.4)	8.1 (7.7)	92.1	90.7	4.2	3.7	81.8 (91.5)	104 (99)
	D	8.0	8.0	0.1	0.5	7.1 (7.4)	8.5 (8.4)	90.8	90.6	4.3	8.0	72.7 (83.1)	109 (104)

Supplementary Table 5. Descriptive statistics calculated for dissolved inorganic carbon (DIC) and dissolved organic carbon (DOC) concentrations using data at per-minute resolution in the estuary types (est. type; LA: lagoons, SD: small deltas, and TS: tidal systems), disturbance (dist.) groups A: low, B: moderate, C: high, and D: very high), and disturbance groups within estuary types. SE: standard error; IQR: interquartile range (3rd quartile-1st quartile). The mean of minimum and maximum values calculated for each estuary are presented in brackets.

		DIC concentration (μmol l^{-1})						DOC concentration (mmol l^{-1})					
Estuary type	Dist. group	Mean	Median	SE	IQR	Min	Max	Mean	Median	SE	IQR	Min	Max
LA		1794.0	1900.7	113.3	395.1	351 (1583)	3455 (2019)	0.556	0.424	0.093	0.462	0.049 (0.486)	2.27 (0.834)
SD		1865.4	2006.8	118.9	421.3	551 (1392)	2435 (2102)	0.319	0.319	0.046	0.199	0.041 (0.138)	1.009 (0.468)
TS		2177.4	2124.0	135.2	256.8	790 (2093)	4677 (2577)	0.204	0.185	0.033	0.178	0.02 (0.126)	0.517 (0.322)
	A	2070.1	2065.7	337.2	294.6	351 (1870)	4677 (2284)	0.408	0.152	0.189	0.464	0.02 (0.509)	1.75 (0.725)
	B	2007.5	2072.9	137.9	344.6	740 (1851)	3287 (2299)	0.376	0.279	0.116	0.325	0.021 (0.377)	2.27 (0.682)
	C	1903.0	1975.8	101.3	370.0	551 (1633)	2435 (2090)	0.322	0.308	0.052	0.223	0.041 (0.168)	1.143 (0.449)
	D	1887.6	2075.4	119.6	443.9	587 (1531)	3455 (2215)	0.361	0.318	0.053	0.206	0.043 (0.223)	1.074 (0.57)
LA	A	1062.5	720.3	448.7	1398.8	351 (913)	2411 (1289)	1.197	1.166	0.211	0.598	0.719 (1.055)	1.75 (1.346)
	B	1772.5	1922.1	149.4	454.5	740 (1770)	2180 (2038)	0.629	0.459	0.187	0.374	0.049 (0.594)	2.27 (0.912)
	C	1822.2	1858.2	122.2	211.5	909 (1591)	2130 (2028)	0.389	0.266	0.125	0.355	0.093 (0.239)	1.143 (0.563)
	D	1999.9	2075.5	223.6	433.6	790 (1722)	3455 (2358)	0.452	0.380	0.111	0.373	0.062 (0.28)	1.074 (0.714)
SD	A	1887.0	1990.0	158.6	373.7	551 (1506)	2435 (2106)	0.315	0.338	0.061	0.200	0.041 (0.127)	0.607 (0.426)
	B	1831.5	2075.8	182.8	570.3	587 (1278)	2286 (2098)	0.324	0.306	0.072	0.180	0.043 (0.149)	1.009 (0.511)
TS	A	2406.0	2126.6	322.9	450.1	2023 (2588)	4677 (3031)	0.145	0.125	0.044	0.110	0.02 (0.099)	0.417 (0.26)
	B	2167.7	2139.8	208.4	329.1	790 (1948)	3287 (2613)	0.180	0.129	0.054	0.192	0.021 (0.074)	0.517 (0.359)
	C	2255.3	2305.5	80.9	78.5	2017 (2123)	2349 (2199)	0.195	0.201	0.041	0.095	0.097 (0.115)	0.271 (0.235)
	D	1877.4	2024.3	202.5	337.1	1010 (1656)	2198 (2163)	0.330	0.336	0.057	0.154	0.117 (0.256)	0.449 (0.399)

Supplementary Table 6. Criteria for assessing disturbance intensity used to classify Australian estuaries. Modified from NLWRA[1] and Borja et al.[2].

**Criteria**	**Condition**			
	**Low**	**Moderate**	**High**	**Very high**
**Catchment natural cover**	> 90%	65 – 90%	< 60%	< 35%
**Land-use**	Limited roads and disturbance to natural conditions and processes	No known gross impacts from land-use (e.g. sediments into waterways and estuary)	Documented impacts from land-use (e.g. sediments and nutrients to waterways)	Documented impacts from land-use throughout waterways and into the estuary
**Catchment hydrology**	No dams or impoundments, virtually nil abstraction	No dams or significant impoundments, some abstraction	Dams and impoundments, significant abstraction modifying natural flows	Dams and impoundments, significant abstraction modifying natural flows
**Tidal regime**	No impediments to tidal flow and changes from natural morphology (e.g. training walls, barrages, bridges, and causeways)	No significant impediments to tidal flow or changes from natural morphology	Impediments to tidal flow and/or changes from natural morphology (e.g. training walls, causeways, and artificial openings of entrance	Major changes to tidal flow and/or major changes from natural morphology
**Floodplain**	Wetlands intact in vegetation and hydrology, with no alterations to flood pattern	Wetlands mostly intact in vegetation and hydrology, with no alterations to flood pattern	Wetlands mostly cleared in vegetation and/or changes in hydrology (e.g. drains, tidal barrages, and levees)	Wetlands mostly cleared in vegetation and/or changes in hydrology (e.g. major losses in fresh to brackish wetlands)
**Estuary use**	Extractive activities limited to indigenous or sustainable commercial and recreational fishing and no aquaculture	Extractive activities limited to sustainable commercial and recreational fishing and minor aquaculture	Extractive activities include dredging, extensive aquaculture, and habitat modifying fishing methods (e.g. prawn trawling)	Extractive activities include dredging, extensive aquaculture, and habitat modifying fishing methods (e.g. prawn trawling)
**Pest and weeds**	Minimal impact on the estuary from catchment weeds and limited pest and weeds within the estuary	Minimal impact on the estuary from catchment weeds and limited pests and weeds within the estuary	Significant impact on the estuary from catchment weeds and impact on estuary ecology from pests and weeds within the estuary	Significant impact on the estuary from catchment weeds and impact on estuary ecology from pests and weeds within the estuary
**Estuarine ecology**	Ecological systems and processes intact (e.g. benthic flora and fauna)	Ecological systems and processes mostly intact (e.g. some changes to benthic flora and fauna)	Ecological systems and processes modified (e.g. loss of benthic flora and fauna)	Ecological systems and processes degraded (e.g. major changes to habitats or species assemblages)

Supplementary Table 7. Conceptual definitions of the three estuary types used to classify Australian estuaries[3].

**Estuary type**	**Conceptual definitions**
Lagoons	- Shallow water bodies (generally <5m deep) separated from the ocean by a barrier.<br>- Commonly elongated and parallel to the coast due to the dominating influence of coastal currents over riverine flow.<br>- Characterised by long residence times and calm waters.<br>- Can also be enclosed shallow water bodies between the river and the coast with minimal tidal influence.
Small deltas	- Formed by sedimentation at the river mouth.<br>- Sediments not removed by tides and/or waves.
Tidal systems	- Strong tidal influence.<br>- No sedimentation at the mouth.<br>- Classic funnel-shaped estuaries usually with long residence times.

Supplementary Figures

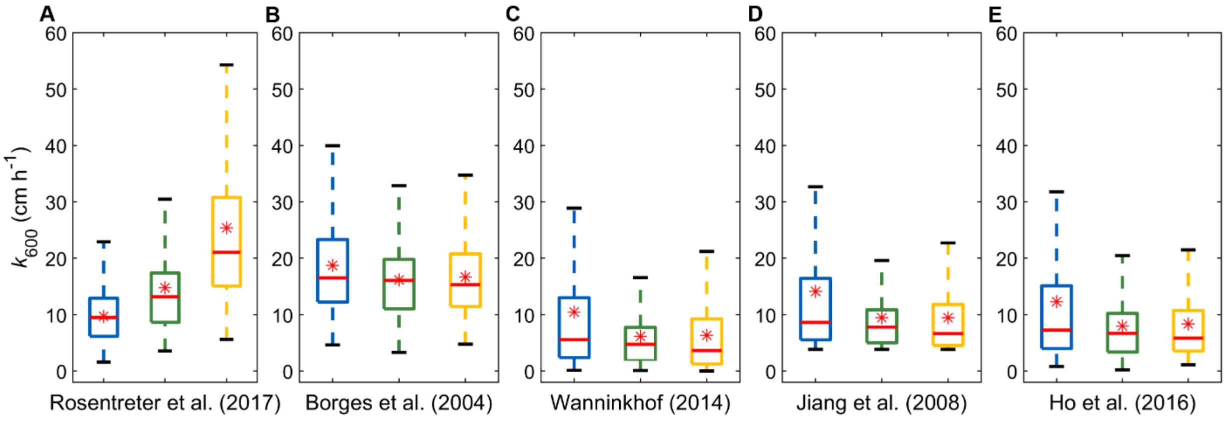

Supplementary Figure 1. Gas transfer velocities calculated using the five parameterisations. Median (red line), mean (red asterisk), 1st and 3rd interquartile ranges (box caps), minimum, and maximum values (whiskers) of gas transfer velocity normalised to Schmidt number 600 (k_{600}) calculated from the five parameterisations (A-E) in the lagoon (blue, n=3789), small delta (green, n=3622) and tidal system (yellow, n=5720) estuary types. Outliers were omitted from the graphs. Source data are provided in the Source Data file.

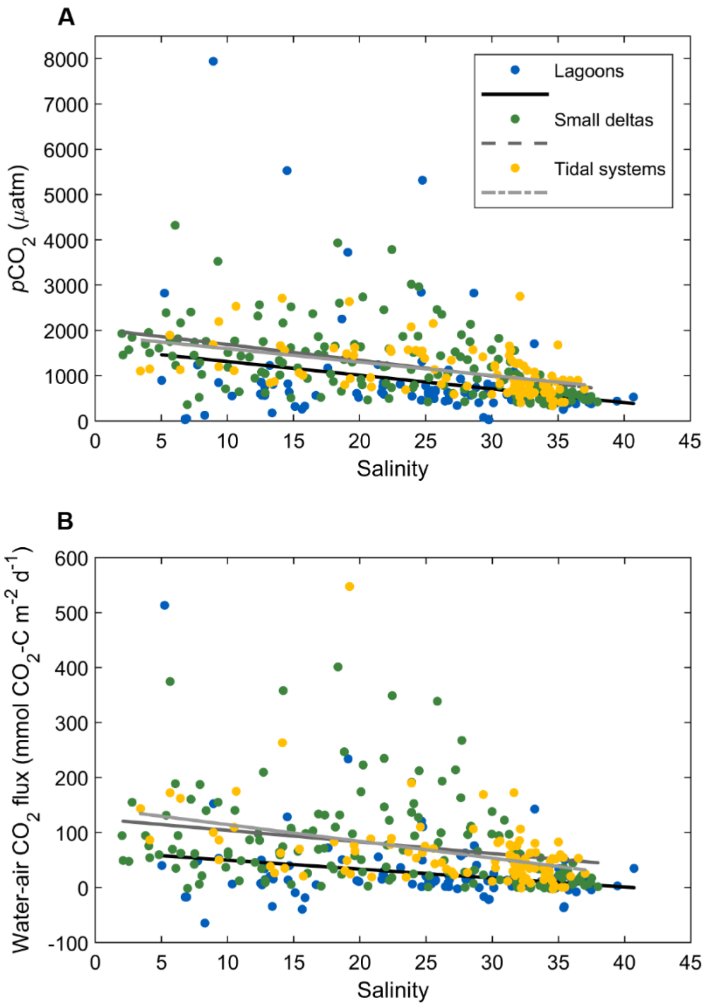

Supplementary Figure 2. Linear relationships between salinity and CO_2. Line of best-fit between salinity and (A) pCO_2 and (B) water-air CO_2 flux in lagoons (blue dots and dark grey solid line, pCO_2: y = -30.19x + 1614.5, r^2 = 0.0629; flux: y = -1.63x + 65.94, r^2 = 0.0578), small deltas (green dots and grey dashed line, pCO_2: y = -34.7x + 2035.1, r^2 = 0.2177;

flux: y = -2.11x + 124.99, r^2 = 0.0728), and tidal systems (yellow dots and light grey dash dotted line, pCO_2: y = -29.52x + 1890.4, r^2 = 0.2361; flux: y = -3.04x + 144.68, r^2 = 0.1533) at 8 km or 5 salinity change resolution (except for the intermittently closed or open lakes and lagoons (ICOLLs), Supplementary Data 1). Source data are provided as a Source Data file.

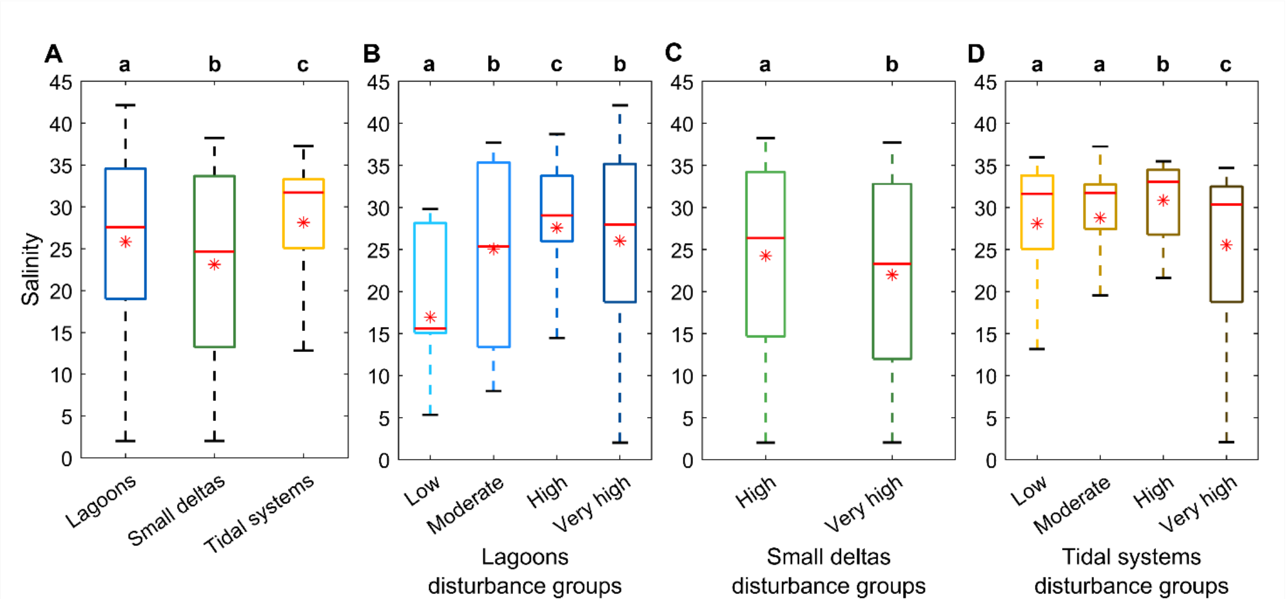

Supplementary Figure 3. Water salinity in the estuary types and estuary type disturbance groups. Median (red line), mean (red asterisk), 1st and 3rd interquartile ranges (box caps), minimum and maximum values (whiskers) of salinity at per-minute resolution across (A) estuary types (n: lagoons (blue)=3789, small deltas (green)=3622, and tidal systems (yellow)=5720, p=0.001) and (B, C, and, D) between disturbance groups in lagoons (light blue to dark blue), n: low=214, moderate=815, high=1312, and very high=1448), small deltas (light green to dark green, n: high=1777 and very high=1845), and tidal systems (yellow to dark brown, n: low=1582, moderate=2374, high=588, and very high=1176). Outliers were omitted from the figures. Letters above figures denote statistical differences among estuary types, with letters that are the same indicating no significant difference (PERMANOVA, two-tailed, and at 95% confidence interval). Source data are provided in the Source Data file.

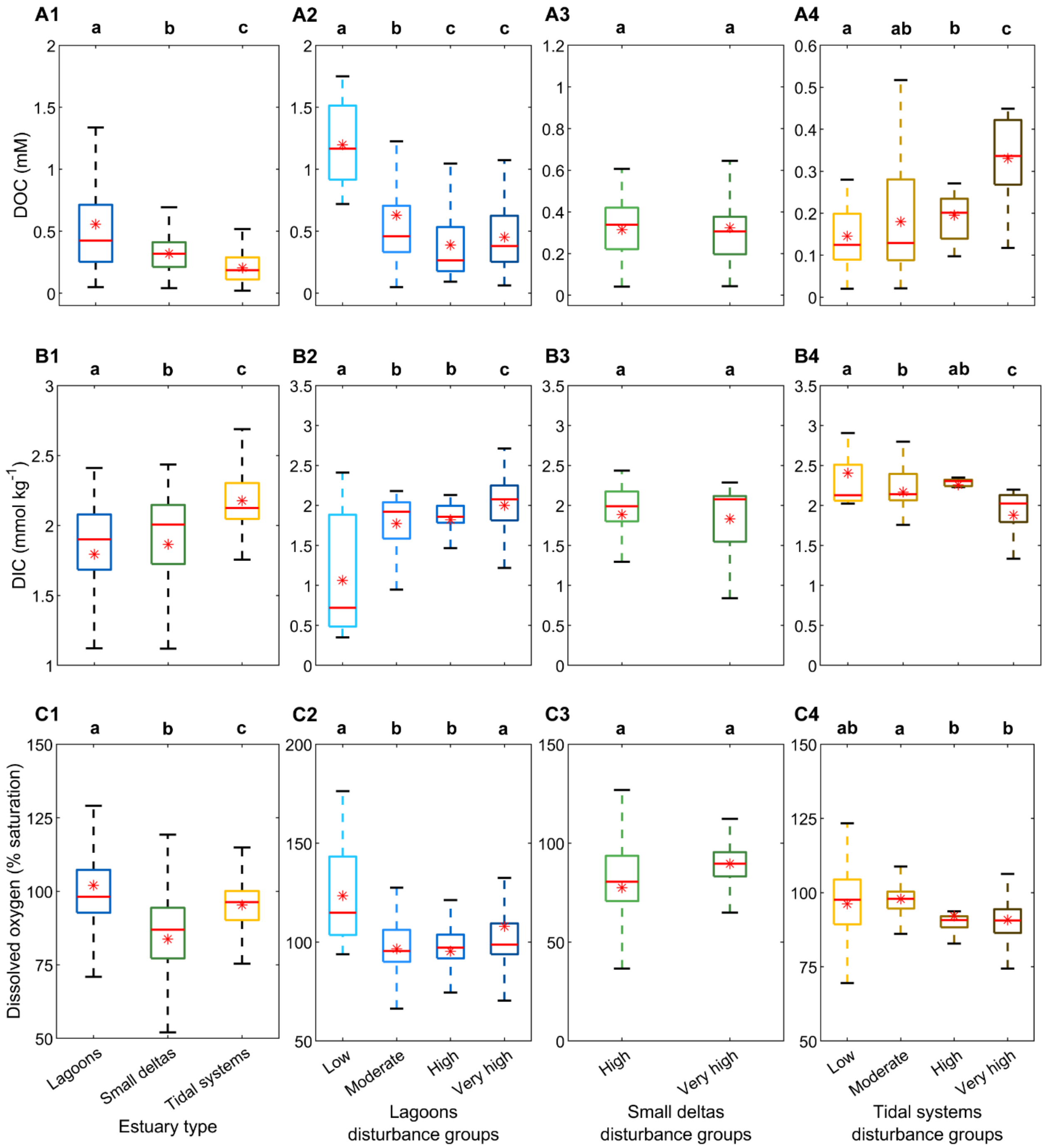

Supplementary Figure 4. Dissolved organic and inorganic carbon and dissolved oxygen in the estuary types and estuary type disturbance groups. Median (red line), mean (red asterisk), 1st and 3rd interquartile ranges (box caps), minimum and maximum values (whiskers) of (row A) dissolved organic carbon (DOC), (row B) dissolved inorganic carbon (DIC), and (row C) dissolved oxygen (%sat.) across (column 1) estuary types (estuary type n=3789, 3622, and 5720, respectively for DOC, DIC, and dissolved oxygen, all p=0.001) and (columns 2, 3, and 4) between disturbance groups (lighter to darker colors, disturbance n=815, 214, 1312, and 1448, respectively, for DOC, DIC, and dissolved oxygen) in each estuary type (lagoons: blue, small deltas: green, and tidal systems: yellow). Outliers were omitted from the figures. Letters above figures denote statistical differences among estuary types, with letters that are the same indicating no significant difference (PERMANOVA, two-tailed, and at 95% confidence interval). Note the different scale on the y-axes. Source data are provided in the Source Data file.

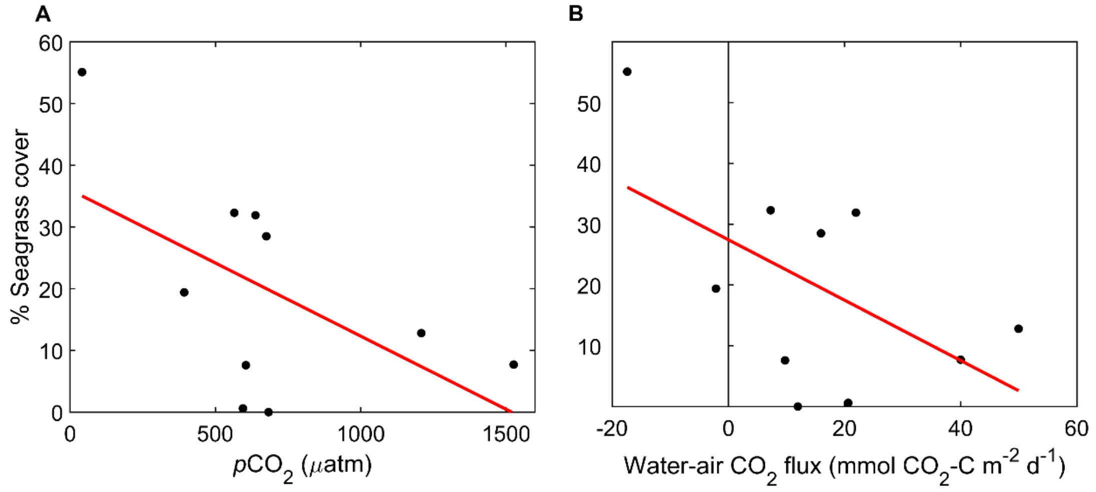

Supplementary Figure 5. Linear relationships between seagrass cover and CO_2. Significant correlations (Partial correlation, two-tailed, and at 95% confidence interval) between per-estuary mean percent seagrass cover and (A) pCO_2 (r=-0.6, p=0.001) and (B) water-air CO_2 flux (r=-0.352, p=0.018) in New South Wales lagoons. Source data are provided as a Source Data file.

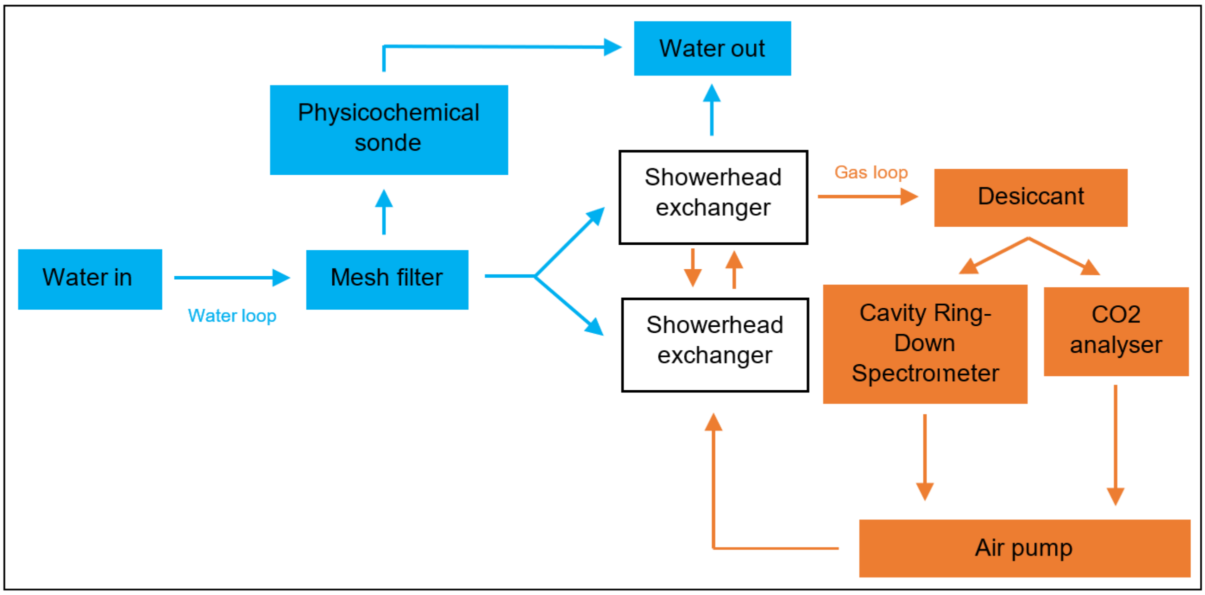

Supplementary Figure 6. Schematic diagram showing the integrated water (blue) and gas (orange) loops for continuous instrument analysis of physicochemical parameters and pCO_2.

Supplementary References

1. NLWRA. *Australian Catchment, River and Estuary Assessment 2002*. vol. 1 (National Land and Water Resources Audit, Commonwealth Government, 2002).

2. Borja, A. *et al.* Classifying Ecological Quality and Integrity of Estuaries. in *Treatise on Estuarine and Coastal Science* (eds. Wolanski, E. & McLusky, D.) vol. 1 125–162 (Waltham: Academic Press, 2012).

3. Dürr, H. H. *et al.* Worldwide typology of nearshore coastal systems: Defining the estuarine

filter of river inputs to the oceans. *Estuaries and Coasts* **34**, 441–458 (2011).
