# Supplementary material for: High carbon dioxide emissions from Australian estuaries driven by geomorphology and climate
Source: Nat Commun. 2024 May 10;15:3967. doi: 10.1038/s41467-024-48178-4 (PMC11087516; doi:10.1038/s41467-024-48178-4)
Supplement: Supplementary file 3 — Description of Additional Supplementary Files [file 41467_2024_48178_MOESM3_ESM.pdf]

<u>Description of Additional Supplementary Files</u>

Supplementary Data 1. Location, survey date, intermittently closed or open lakes and lagoons (ICOLL) status, water area, and estuary type and disturbance classifications of the surveyed estuaries. *denotes estuaries that were surveyed with a smaller boat rather than the main research boat (see Methods section).

Supplementary Data 2. Descriptive statistics for pH and dissolved oxygen in each sampled estuary, inclusive of percent cleared catchment land, estuary type, and disturbance classifications. Interquartile ranges were calculated by subtracting the 3rd quartile with the 1st quartile.

Supplementary Data 3. The state, geographical coordinates, audit number as in the National Land and Water Resources Audit (NLWRA), estuary type (LA: lagoons, SD: small deltas, and TS: tidal systems) and disturbance (disturbance groups: A: low, B: moderate, C: high, and D: very high) classifications, tidal range, catchment area, and water area for all of Australia's estuaries. *denotes water areas calculated in this study.
