# Supplementary material for: High carbon dioxide emissions from Australian estuaries driven by geomorphology and climate
Source: Nat Commun. 2024 May 10;15:3967. doi: 10.1038/s41467-024-48178-4 (PMC11087516; doi:10.1038/s41467-024-48178-4)
Supplement: Supplementary file 7 — Reporting Summary [file 41467_2024_48178_MOESM7_ESM.pdf]

nature portfolio

Corresponding author(s): Jacob Yeo

Last updated by author(s): Mar 1, 2024

Reporting Summary

Nature Portfolio wishes to improve the reproducibility of the work that we publish. This form provides structure for consistency and transparency in reporting. For further information on Nature Portfolio policies, see our Editorial Policies and the Editorial Policy Checklist.

Statistics

For all statistical analyses, confirm that the following items are present in the figure legend, table legend, main text, or Methods section.

n/a	Confirmed	
☐	☒	The exact sample size (*n*) for each experimental group/condition, given as a discrete number and unit of measurement
☐	☒	A statement on whether measurements were taken from distinct samples or whether the same sample was measured repeatedly
☐	☒	The statistical test(s) used AND whether they are one- or two-sided<br>*Only common tests should be described solely by name; describe more complex techniques in the Methods section.*
☐	☒	A description of all covariates tested
☐	☒	A description of any assumptions or corrections, such as tests of normality and adjustment for multiple comparisons
☐	☒	A full description of the statistical parameters including central tendency (e.g. means) or other basic estimates (e.g. regression coefficient) AND variation (e.g. standard deviation) or associated estimates of uncertainty (e.g. confidence intervals)
☐	☒	For null hypothesis testing, the test statistic (e.g. *F*, *t*, *r*) with confidence intervals, effect sizes, degrees of freedom and *P* value noted<br>*Give P values as exact values whenever suitable.*
☒	☐	For Bayesian analysis, information on the choice of priors and Markov chain Monte Carlo settings
☐	☒	For hierarchical and complex designs, identification of the appropriate level for tests and full reporting of outcomes
☐	☒	Estimates of effect sizes (e.g. Cohen's *d*, Pearson's *r*), indicating how they were calculated

Our web collection on statistics for biologists contains articles on many of the points above.

Software and code

Policy information about availability of computer code

Data collection	No coding software was used for data collection. Data organisation and processing was carried out using Microsoft Excel and Matlab.
Data analysis	Statistical analyses were performed using IBM SPSS version 29 for pearsons and partial correlations and Primer v7 with PERMANOVA+ for PERMANOVA analyses. Plots and linear correlations were generated using Matlab. Maps and GIS measurements were done using Google Earth and QGIS 3.22.

For manuscripts utilizing custom algorithms or software that are central to the research but not yet described in published literature, software must be made available to editors and reviewers. We strongly encourage code deposition in a community repository (e.g. GitHub). See the Nature Portfolio guidelines for submitting code & software for further information.

Data

Policy information about availability of data

All manuscripts must include a data availability statement. This statement should provide the following information, where applicable:

- Accession codes, unique identifiers, or web links for publicly available datasets
- A description of any restrictions on data availability
- For clinical datasets or third party data, please ensure that the statement adheres to our policy

The environmental survey data generated/used in this study is freely available and has been deposited in the FigShare database under accession code 10.6084/m9.figshare.25242676. Figure source data are provided with this paper as part of the Supplementary Information.

Research involving human participants, their data, or biological material

Policy information about studies with human participants or human data. See also policy information about sex, gender (identity/presentation), and sexual orientation and race, ethnicity and racism.

Reporting on sex and gender	NA
Reporting on race, ethnicity, or other socially relevant groupings	NA
Population characteristics	NA
Recruitment	NA
Ethics oversight	NA

Note that full information on the approval of the study protocol must also be provided in the manuscript.

Field-specific reporting

Please select the one below that is the best fit for your research. If you are not sure, read the appropriate sections before making your selection.

☐ Life sciences ☐ Behavioural & social sciences ☒ Ecological, evolutionary & environmental sciences

For a reference copy of the document with all sections, see nature.com/documents/nr-reporting-summary-flat.pdf

Ecological, evolutionary & environmental sciences study design

All studies must disclose on these points even when the disclosure is negative.

Study description	The study estimates the annual CO2 emission from all Australian estuaries from a sample set of 47 estuaries. Differences in CO2 emissions from estuaries of 3 types of estuarine geomorphology and 4 levels of anthropogenic disturbance were assessed, including disturbance effects within each estuary type, in a nested factorial design. The number of estuaries studied in each group is indicated in Table 3, in which per-minute instrumental measurement for pCO2 and discrete water samples for nutrient and carbonate chemistry were taken every hour or at 5 salinity intervals. Water-air CO2 flux rates were calculated from the measured pCO2 and then upscaled to each classification level, and then to all of Australian estuaries. The amount of replications in each factor group is reported in Table 3.
Research sample	47 estuaries were chosen to give a representative set of estuaries of different estuary types and disturbance levels while covering a wide spatial distribution around Australia, accounting for different climatic and geographic areas. As a wide spatial coverage is the study aim in order to give a overall representation of CO2 emissions from Australian estuaries, only summer measurements were taken. 36 estuaries were surveyed as part of this study's fieldwork campaign, with 11 previously published estuaries also included. These studies are referenced in the Methods section.
Sampling strategy	High resolution, per-minute measurements of CO2 and physiochemical data from the 47 sample estuaries were measured along the salinity transect from the mouth to the freshwater reaches (>2 salinity) or where further progress was impeded by obstacles. Discrete samples for carbonate chemistry were also taken along the survey transects at 1 hour intervals or at 5 salinity changes, whichever occurs first. The number of sample estuaries were based on the proportion of surface area coverage of each estuary type across the Australian continent, as reported in Table 3. This survey represented 12.3% of all estuarine surface area across Australia.
Data collection	Collection of pCO2 (Picarro G2308 and LiCOR Li-840A), physiochemical (Hydrolab HL4), meteorological (Airmar 200WX), and physical data was recorded via instruments onboard the survey vessel as described in the Methods section. Discrete samples for chemical analysis were taken during the survey and taken back to the lab for lab-based analysis as described in the Methods Section. Jacob Yeo was the fieldwork lead on the project and collected all data with the help with field volunteers. Co-author contributions can be found in the Author Contribution Statement.
Timing and spatial scale	This information is provided in Supplmentary Table 6 of the manuscript. Overall, samples were taken over two subsequent summers (2017 and 2018) as it would have been difficult to complete the fieldwork campaign over one summer season. Summer was chosen in order to produce a conservative estimate, driven by higher summer temperatures. Sample estuaries cover the southwestern, eastern, and northern Australian coasts, accounting for geographical differences across the continent. Winter flux rates were calculated using seasonal factors derived from estuaries with available winter and summer data. Mean annual flux rates were then calculated with summer and winter flux rates and used to upscale to all of Australian estuaries.
Data exclusions	Seagrass coverage data from Termeil and Lake Brou were excluded from seagrass coverage analysis due to inconsistent database data relative to on-ground validation findings, as indicated in the Methods.
Reproducibility	This study was carried out using environmental survey data where conditions were not controlled. Surveys were done once and were not repeated based on any bias. The estuarine surveys were done in similar daylight conditions, following the same upstream

	transect methodology starting at high tide at the river mouth. When weather conditions were forecasted to be adverse (heavy rain) at the start or during the survey, fieldwork was postponed to the next available day with good weather conditions.
Randomization	Samples are grouped according to specific classification criteria and were therefore not randomised.
Blinding	No blinding strategy

Did the study involve field work? ☒ Yes ☐ No

Field work, collection and transport

Field conditions	The estuary surveys covered a wide spatial scale and were representative of typical conditions of the estuaries. Individual estuary information can be found in Supplementary Table 5. Detailed meteorological data on the survey dates are available at the Australian Bureau of Meteorology database (http://www.bom.gov.au/climate/data/index.shtml?bookmark=136&zoom=3&lat=-32.5355&lon=147.74&layers=B00000TFFFFFFFTFFFFFFFFFFFFFFFFFFFFFFFFTTT&dp=IDC10002-d).
Location	This information is provided within Supplementary Table 5 in the manuscript, with surveys covering the east, north, and southwest coasts of Australia.
Access & import/export	Estuaries were accessed after completing any required approval and paperwork. A permit was obtained for the South Alligator River survey (PA2018-00054).
Disturbance	This study had minimal disturbance to the environment as only small quantities of surface water samples were taken. No chemicals were added into the environment. No flora and fauna were taken from the environment.

Reporting for specific materials, systems and methods

We require information from authors about some types of materials, experimental systems and methods used in many studies. Here, indicate whether each material, system or method listed is relevant to your study. If you are not sure if a list item applies to your research, read the appropriate section before selecting a response.

Materials & experimental systems

n/a	Involved in the study
☒	☐ Antibodies
☒	☐ Eukaryotic cell lines
☒	☐ Palaeontology and archaeology
☒	☐ Animals and other organisms
☒	☐ Clinical data
☒	☐ Dual use research of concern
☒	☐ Plants

Methods

n/a	Involved in the study
☒	☐ ChIP-seq
☒	☐ Flow cytometry
☒	☐ MRI-based neuroimaging
